# The mechanical niche in gastric precancer: extracellular matrix remodeling, mechanotransduction, and stem-cell fate

**DOI:** 10.3389/fcell.2026.1970363

**Published:** 2026-09-28

**Authors:** Lanshuo Hu, Xuanchun Huang, Tai Zhang, Yeqian Wu, Liangjun Yang, Jiajie Zhu, Xudong Tang, Baoying Fei

**Affiliations:** 1 Department of Gastroenterology, Tongde Hospital of Zhejiang Province, Hangzhou, China; 2 The First Affiliated Hospital of Zhejiang Chinese Medical University, Hangzhou, China; 3 Zhejiang Provincial Hospital of Traditional Chinese Medicine, Hangzhou, China; 4 Peking University Traditional Chinese Medicine Clinical Medical School (Xiyuan), Peking University Health Science Center, Beijing, China; 5 Peking University Health Science Center, Beijing, China; 6 Institute of Digestive Diseases, Xiyuan Hospital, China Academy of Chinese Medical Sciences, Beijing, China; 7 Tongde Hospital of Zhejiang Province Affiliated to Zhejiang Chinese Medical University (College of Integrated Traditional Chinese and Western Medicine Clinical Medicine), Hangzhou, China

**Keywords:** dynamic loading, extracellular matrix, gastric precancerous lesions, GPMES, mechanotransduction, Piezo1, stem-cell plasticity, YAP/TAZ

## Abstract

**Background:**

The Correa cascade foregrounds infection, inflammation, and epithelial change but does not fully explain how the physical tissue environment influences progression from atrophy to metaplasia and dysplasia.

**Methods:**

We integrated human observations, gastric organoid and animal perturbation studies, advanced gastric-cancer experiments, and cross-tissue evidence using the Gastric Precancer Mechanobiology Evidence Stratification framework, which ranks evidence by proximity to causal human precancerous mechanics.

**Results:**

Direct human mechanical measurements remain sparse and methodologically heterogeneous, precluding a universal stiffening trajectory. YAP has the strongest functional support across gastric regeneration and metaplastic remodeling, integrin–adhesion signaling is convergent but mechanically under-specified, and Piezo1 evidence derives mainly from advanced cancer. Three non-exclusive models—mechanical driver, epithelial-first remodeling, and inflammation-mediated feedback—produce distinct temporal predictions.

**Conclusion:**

Gastric precancer is best understood through a mechanically extended Correa framework in which static matrix properties, dynamic loading, regional anatomy, inflammatory context, and lineage state jointly determine whether injury resolves or stabilizes abnormal epithelial states. Spatial human mechanics linked to dynamic patient-derived perturbation models are required before mechanical biomarkers or pathway-directed interventions can guide clinical care.

## 1 Introduction

### 1.1 Beyond a linear correa cascade: gastric precancer as a branching tissue-state transition

Gastric carcinogenesis is commonly organized around the Correa cascade, in which chronic mucosal injury is followed by atrophy, metaplasia, dysplasia, and ultimately invasive cancer. This sequence remains clinically useful because it identifies a prolonged window during which tissue trajectories may still be monitored or intercepted. Yet, it should not be interpreted as an obligatory linear relay. Gastric epithelial populations are regionally heterogeneous, differentiated cells can reacquire plasticity after injury, and mutated stem-cell clones may expand through field cancerization. Consequently, spasmolytic polypeptide-expressing metaplasia (SPEM), intestinal metaplasia, and dysplasia may arise through partially overlapping or parallel routes rather than through a single universal lineage sequence (McDonald et al., 2008; Zhang and Tang, 2026; Zhang et al., 2026).

This revised view shifts attention from isolated molecular markers to the tissue conditions that determine whether an injured gland restores normal architecture or stabilizes an abnormal lineage state. Gastric renewal depends on region-specific stem and progenitor populations, differentiated epithelial cells, stromal and immune cells, extracellular matrix (ECM), and soluble niche signals. Experimental atrophic gastritis models show that impaired progenitor maintenance accompanies loss of differentiated lineages and defective regeneration, whereas restoration of selected niche signals can improve regenerative output (Li K. et al., 2024). The central question is therefore not simply which markers accompany atrophy or metaplasia, but how the damaged mucosa integrates biochemical and physical information during repeated attempts at repair.

### 1.2 The mechanical niche as a regulatory layer in gastric epithelial homeostasis

Most models of gastric precancer emphasize infection, inflammation, somatic alterations, and biochemical signaling. These mechanisms are indispensable, but they operate within a physical tissue whose material and structural properties change during chronic injury. The ECM is not merely a passive scaffold. Its composition, density, organization, and coupling to cell–cell and cell–matrix adhesions determine how forces are transmitted across glands and converted into intracellular signals. Chronic gastritis is associated with altered matrix-remodeling networks, including metalloproteases, their inhibitors, and disintegrin-metalloproteases (Sampieri, 2013). Premalignant gastric tissues also exhibit detectable alterations in gland morphology and collagen-associated optical signals, although such imaging readouts characterize tissue architecture rather than directly measuring stiffness (Li et al., 2018; Li et al., 2019).

The term mechanical niche is used here to describe the integrated system formed by ECM composition and architecture, basement-membrane and adhesion complexes, tissue material properties, cellular tension, and the molecular machinery that senses and transduces physical inputs. This definition deliberately separates four quantities that are often conflated: ECM abundance, fiber organization, ligand density, and tissue stiffness. For example, increasing collagen-matrix density can disrupt the E-cadherin/β-catenin complex and alter focal-adhesion signaling in gastric cancer cells (Jang et al., 2018), but this observation does not by itself establish that all gastric precancerous lesions are mechanically stiffer. Likewise, changes detected by second-harmonic generation or multimodal nonlinear optical microscopy provide valuable structural evidence but are not equivalent to atomic-force microscopy, indentation, or rheological measurements (Li et al., 2018; Li et al., 2019).

The aim of this review is to explain how regional gland architecture and extracellular-matrix organization condition epithelial behavior during gastric precancer, while separating three levels of inference that are often conflated: observed tissue remodeling, involvement of a mechanosensitive pathway, and causality attributable to a defined physical input. We address three linked questions. First, how do corpus and antral gland geometry, lineage organization, and matrix interfaces establish different starting niches? Second, which matrix changes have been observed across atrophy, metaplasia, and dysplasia, and which material properties remain unmeasured? Third, when do integrin–FAK/Src, YAP/TAZ, and Piezo1 data support pathway function rather than direct mechanical causality? The review follows that order—from regional anatomy and ECM organization (Section 2), through stage-dependent remodeling (Section 3) and mechanotransduction (Section 4), to lineage outcomes (Section 5), multicellular feedback (Section 6), and causal and translational tests (Sections 7 and 8). GPMES is used throughout to keep the evidentiary boundary visible at each transition.

### 1.3 Mechanotransduction links tissue remodeling to epithelial fate decisions

Several evidence streams make a mechanobiological synthesis timely. First, cell–matrix adhesion pathways are altered during early or intestinal-type gastric carcinogenesis. The collagen receptor ITGA2 is regulated by miR-30a in models spanning metaplasia and early gastric cancer, while recurrent CLDN18–ARHGAP26 fusion disrupts epithelial integrity and RHOA-related behavior (Min et al., 2020; Yao et al., 2015). These studies connect adhesion and polarity to disease progression, although they do not independently prove that a defined mechanical stimulus is responsible for the observed phenotype.

Second, the Hippo pathway provides an interface between tissue architecture, cytoskeletal state, and epithelial plasticity. Gastric studies link LATS2 and YAP-centered programs to H. pylori-associated epithelial responses, oxyntic-gland regeneration, and metaplastic reprogramming (Fischer et al., 2022; Loe et al., 2023; Molina-Castro et al., 2020). These findings make YAP a useful example of why pathway activity must be interpreted by tissue compartment, disease stage, and experimental context. Nuclear localization alone is therefore not a stage-independent marker of pathogenic mechanical activation; the regulatory mechanisms and lineage consequences are examined separately in Sections 4 and 5.

Third, Piezo1 offers a candidate molecular sensor that can link physical perturbation to calcium-dependent responses. Functional manipulation of Piezo1 in gastric cancer models connected the channel to Ca^2+^ influx, HIF-1α-associated signaling, epithelial–mesenchymal transition, angiogenic programs, and omental metastasis (Wang et al., 2021). This evidence is mechanistically relevant but derives primarily from advanced disease and does not establish Piezo1 function during atrophy, metaplasia, or dysplasia. Taken together, integrin–FAK, cytoskeletal–YAP/TAZ, and Piezo1 pathways support a coherent model of mechanical signal integration, while simultaneously illustrating how much of the current mechanistic evidence must still be validated in the precancerous setting (Fischer et al., 2022; Jang et al., 2018; Loe et al., 2023; Molina-Castro et al., 2020; Wang et al., 2021; Zhang et al., 2019).

### 1.4 Evidence gaps and the GPMES analytical framework

The central unresolved question is not whether the gastric ECM changes during precancer, but which physical properties change, where they change, and whether those changes alter epithelial fate. Existing studies document matrix composition, collagen organization, adhesion, and mechanotransduction-related pathways, yet spatially registered measurements of stiffness, viscoelasticity, residual stress, and force transmission across normal, atrophic, metaplastic, and dysplastic mucosa remain limited. Current evidence therefore does not support representing gastric precancer as a single monotonic stiffening trajectory (Jang et al., 2018; Li et al., 2018; Li et al., 2019). This wording does not exclude local stiffening; it emphasizes that direction, magnitude, tissue layer, anatomical region, and disease stage have not been established as one general sequence.

A second challenge is causal order. Matrix remodeling may precede epithelial reprogramming, an altered epithelium may recruit secondary stromal remodeling, or chronic inflammation may couple both processes in a reinforcing loop. The same ambiguity applies to YAP and other candidate mechanosensors: pathway activity may participate in repair, stabilize an altered lineage state, or arise downstream of tissue remodeling, depending on the cellular compartment and injury context (Fischer et al., 2022; Loe et al., 2023; Molina-Castro et al., 2020). Resolving these alternatives requires experiments that separate physical inputs from ligand density, soluble signals, and disease-stage effects.

Studies were assigned to GPMES tiers using three criteria: the biological material and disease stage studied, whether a physical property or input was measured or experimentally controlled, and whether the design established association, functional dependence, or mechanical causality. Tier 1 comprises direct human gastric mechanical measurements; Tier 2, human structural or spatial proxies; Tier 3, functional perturbation in gastric organoids or animal models; Tier 4, pathway manipulation in established gastric cancer; and Tier 5, cross-tissue analogy. Assignment reflects proximity to the central question of causal gastric-precancer mechanobiology, not the overall methodological quality or clinical importance of a study. A rigorous Tier 4 experiment may therefore be less directly applicable to precancer than a small Tier 1 feasibility study.

PubMed and Web of Science were searched on 4 August 2026 without a lower publication-date restriction. The search combined terms for gastric atrophy, intestinal metaplasia, dysplasia, and related precancerous lesions with terms for extracellular matrix, collagen, laminin, tissue mechanics, mechanotransduction, YAP/TAZ, integrins, focal adhesion kinase, Piezo1, and gastric stem/progenitor cells. The search identified 10,451 records and 10,401 remained after conservative cross-database deduplication. Title-and-abstract screening retained 166 records for detailed assessment: 69 concerned stem/progenitor transitions, 54 ECM remodeling, and 43 mechanosensing. Studies were selected for citation when they directly supported a claim, clarified a conflicting result, supplied a relevant method, or defined the limits of extrapolation; abstract-only records were not used to support central mechanistic conclusions.

Within this framework, the review has three analytical outputs. GPMES displays how closely each study approaches causal gastric-precancer mechanobiology. The three competing causal models convert disagreements about directionality into testable differences in temporal ordering, sensor dependence, and reversibility. Finally, regional anatomy and dynamic loading are integrated with static matrix properties to specify what must be measured in future experiments. These outputs allow the mechanical niche to be evaluated as a potential driver, permissive context, or consequence of progression without assigning that role in advance. The scope is restricted to the adenocarcinoma-related precancer sequence; type 1 enterochromaffin-like-cell neuroendocrine tumors represent a distinct lesion pathway (Poveda et al., 2023).

## 2 Regional gland architecture and extracellular-matrix organization in the normal stomach

Mechanical abnormalities can be interpreted only against normal gastric organization. Homeostasis is not mechanically static: glands renew epithelial lineages while accommodating luminal pressure, deformation, secretion, and intermittent injury. Stability therefore depends on region-specific epithelial populations coupled to basement membrane, interstitial ECM, stromal and immune cells, and neural and endocrine inputs. This chapter defines that reference state while separating established lineage biology from mechanobiological hypotheses.

### 2.1 Regional epithelial architecture establishes distinct starting conditions

Marker studies are most informative when interpreted within a defined anatomical compartment. In human tissue, LGR5, Musashi-1, CD44, and related candidates show stage- and gland-dependent localization, including enrichment of intestinal stem-cell markers at the base of metaplastic glands (Akasaka et al., 2005; Jang et al., 2015; Liu et al., 2025; Niu et al., 2023; Takada et al., 2023; Wang et al., 2011; Zhang Q. et al., 2026; Zheng et al., 2013). Organoid, flow-cytometric, single-cell, and lineage approaches likewise resolve functionally distinct epithelial subpopulations rather than one interchangeable gastric stem-cell pool (Bockerstett et al., 2018; Matsuo et al., 2017; Min et al., 2022; Simon et al., 2012). The relevant mechanobiological question is therefore which population occupies a particular corpus or antral compartment when injury and matrix remodeling occur, not which marker is universally ‘stem-like.'

Functional perturbations reinforce this context dependence and fall into two broad groups. Epithelial-intrinsic changes—including NAD + depletion, RUNX3 mutation, or activated Ras in defined lineages—alter genome integrity, differentiation, or metaplastic potential (Choi et al., 2016; Douchi et al., 2022; Li Z. et al., 2026; Shibata et al., 2012). Stromal and infection-associated changes—including BMP signaling, *Helicobacter pylori*-dependent mobilization of Lrig1-positive cells, and SDF-1-dependent stromal expansion—modify the niche in which those epithelial states compete (Liu et al., 2026; Wroblewski et al., 2019). Studies of atrophic gastritis, intracellular *H. pylori* in progenitors, and microbial adaptation further show that injury history changes this context (Bessede et al., 2016; Giannakis et al., 2008; Giannakis et al., 2009; Oh et al., 2005; Tsukamoto et al., 2006; Wang et al., 2022). Because region, model, perturbation, and endpoint differ, these findings are compared as alternative injury responses rather than assembled into one lineage hierarchy.

#### 2.1.1 Corpus and antral glands differ in lineage organization and injury response

Corpus and antral mucosa should not be represented by one generic gland. Corpus/fundus units contain relatively short pits and long, straight oxyntic glands. Their proliferative zone lies mainly in the isthmus, above a neck region containing mucous-neck and parietal cells and a base enriched in chief cells; endocrine cells are distributed through the unit. Antral/pyloric units instead have deeper pits and shorter, branched or coiled mucous glands, with abundant mucous cells and gastrin-producing G cells. Progenitor activity has been localized to both isthmal and basal compartments, although the contribution assigned to each compartment depends on species, marker, and injury model (Bockerstett et al., 2018; Matsuo et al., 2017; Zhang T. and Tang, 2026). These differences define distinct epithelial geometries, cell neighborhoods, and regenerative starting states.

These anatomical differences alter both the cellular target of injury and the route available for repair. Parietal-cell loss disrupts the corpus neck–base axis and can mobilize isthmus-associated progenitors or chief-cell plasticity in experimental oxyntic injury. Antral renewal more often involves basal or isthmal progenitor pools embedded within deep-pit, branched glands (Bockerstett et al., 2018; Choi et al., 2016; Matsuo et al., 2017; Nam et al., 2010). The comparison does not imply that gland shape itself causes a tumor. Rather, region defines the epithelial populations, basement-membrane surface, stromal neighborhood, endocrine exposure, and gland geometry through which an injury or mutation is expressed.

Regional anatomy can therefore modify—not predetermine—the path from altered cell state to clonal growth. In mouse models, activated Ras in eR1-marked stem cells and in chief-cell lineages produces metaplastic or neoplastic programs through different cellular contexts; RUNX3 mutation expands corpus isthmus stem cells; and gastrin-dependent Cck2r-positive corpus progenitors can enhance ulcer repair while restraining dysplasia (Choi et al., 2016; Douchi et al., 2022; Matsuo et al., 2017; Zheng et al., 2026). Together, these experiments support an interaction among region, cell of origin, mutation, injury, and niche state, but they do not establish a direct regional mechanical cause of tumor growth. Figure 1A therefore compares corpus/fundus and antral/pyloric units explicitly and labels the illustrated injury and clonal-expansion routes as model-dependent.

**FIGURE 1:**
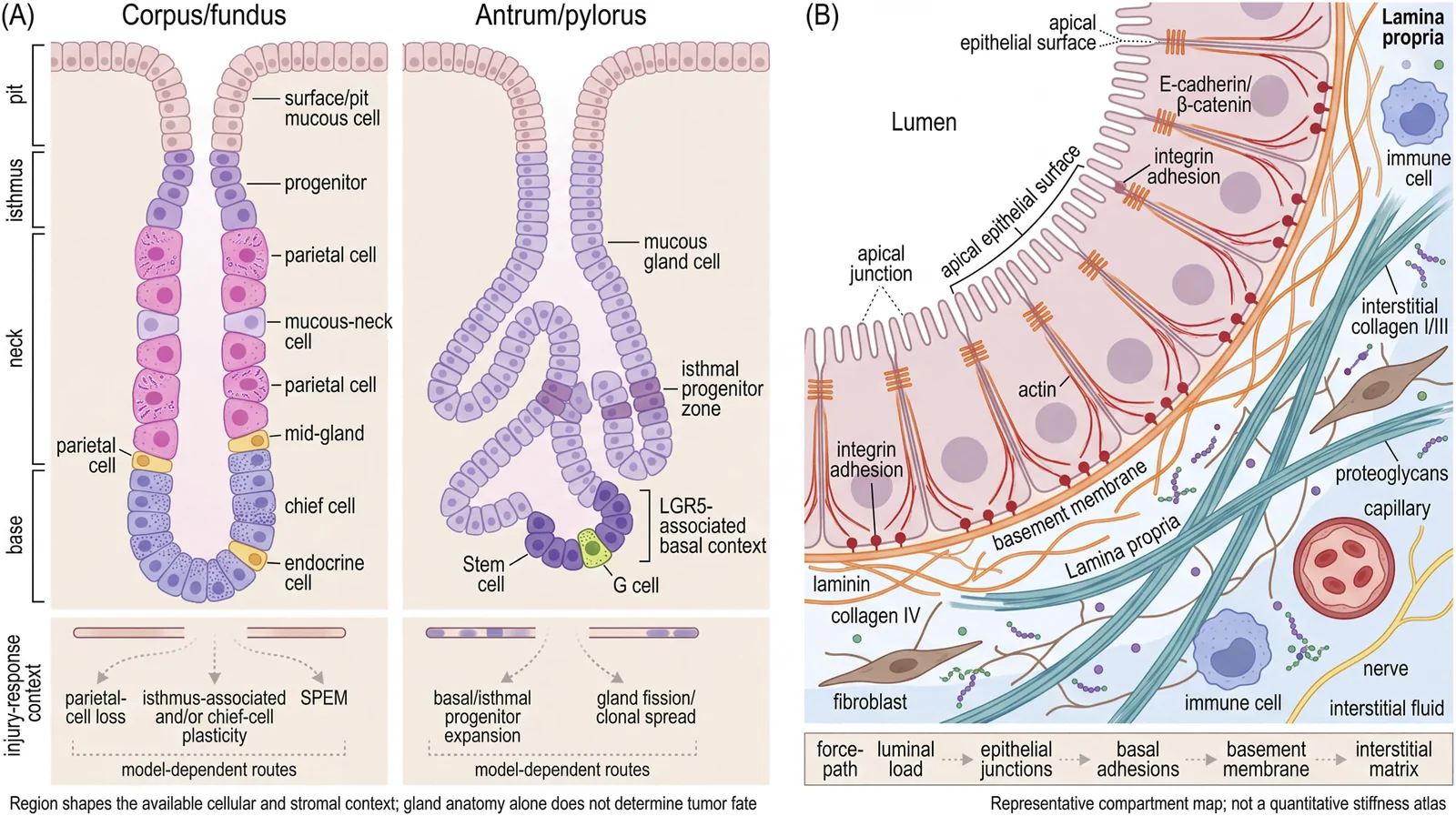
Regional gastric gland architecture and extracellular-matrix organization. **(A)** Corpus/fundus and antral/pyloric gland units are depicted side by side as distinct anatomical systems. Corpus units contain a short pit, an isthmal proliferative compartment, parietal and mucous-neck cells, and a chief-cell-rich base; antral units contain deeper pits, branched or coiled mucous glands, isthmal and basal progenitor contexts, and gastrin-producing G cells. Injury-route insets indicate model-dependent corpus plasticity and antral clonal expansion without implying that gland shape determines tumor fate. **(B)** A magnified gland-wall cross-section is oriented from lumen to epithelium, basement membrane, and lamina propria. It identifies apical and lateral junctions, basal integrin adhesions, laminin and type IV collagen in the basement membrane, and representative interstitial components including collagen I/III, fibronectin, proteoglycans, fibroblasts, immune cells, capillaries, nerves, and interstitial fluid. Proposed force paths are visually separated from established anatomical relationships. The schematic is a compartment map, not a quantitative stiffness atlas (Matsuo et al., 2017; Sampieri, 2013; Tani et al., 1996).

Human mitochondrial lineage analysis shows that gastric units can undergo monoclonal conversion and that mutant clones can spread through gland fission; intestinal-metaplastic crypts likewise display clonal organization and lateral expansion (McDonald et al., 2008). This establishes gland-level selection, not only individual-cell decisions. For mechanobiology, fission is relevant because epithelial packing, basement-membrane boundaries, and surrounding stroma must remodel, although the forces were not measured.

#### 2.1.2 Injury mobilizes lineage-restricted and facultative progenitor programs

After injury, experimental atrophic gastritis models show reduced stem-cell-associated activity and organoid formation, with recovery after restoration of an EGFR-linked niche signal (Li K. et al., 2024). Lineage tracing identifies chief-cell and isthmus-associated contributions under different perturbations (Bockerstett et al., 2018; Nam et al., 2010), while DCAMKL1-positive cells change position during regeneration, metaplasia, and dysplasia (Kikuchi et al., 2010). Gastrin-dependent expansion of Cck2r-positive corpus progenitors improved ulcer repair and restrained dysplasia in one model (Zheng et al., 2026). Regenerative outcome therefore depends on lineage, region, injury model, and signal timing.

Table 1 compares the principal stem/progenitor populations by anatomical and evidentiary context, distinguishing marker expression from lineage function and identifying where mechanical-response evidence is absent.

**TABLE 1:** Gastric stem/progenitor populations, lineage roles, and available mechanical-response evidence.

Marker or population	Region or proposed role	Change or function relevant to precancer	Main evidence model	Principal limitation	References	GPMES tier/mechanical-response interpretation
LGR5-associated cells	Predominantly antral gland renewal; context-dependent injury response	Altered abundance and clonal behavior during atrophy, metaplasia, and carcinogenesis	Genetic lineage studies, human tissue, reviews	Marker and lineage definitions vary across models	(Li K. et al., 2024; McDonald et al., 2008; Zhang T. and Tang, 2026)	Tier 2–3: Not directly defined; regional mechanics unmeasured
AQP5-associated cells	Gastric stem/progenitor compartment in selected models	Reduced stem-cell-associated signal in atrophic gastritis and recovery after niche intervention	Mouse tissue, organoids, human tissue	Clinical sample size and population equivalence require confirmation	(Li K. et al., 2024)	Tier 3: Not directly tested under controlled mechanical loading
Bmi1-associated cells	Reserve or injury-responsive progenitor state	Reduced renewal in experimental atrophy; potential regenerative recruitment	Genetic and organoid evidence	Relationship to other marker-defined populations is context-dependent	(Li K. et al., 2024)	Tier 3: Not directly tested; injury context may alter response
Cck2r-positive corpus progenitors	Corpus proliferative compartment responsive to gastrin	Expansion supports ulcer healing and restrains dysplastic progression in the reported model	Lineage tracing and functional mouse experiments	Recent, model-specific evidence requires independent replication	(Zheng et al., 2026)	Tier 3: No direct gastric force-response data
Chief-cell/SPEM-associated lineage	Oxyntic-gland injury response and metaplastic reprogramming	YAP-associated regenerative program can become sustained during SPEM/metaplasia	Genetic mouse models, single-cell and human validation	Shared repair and metaplasia programs do not imply inevitable cancer progression	(Fischer et al., 2022; Loe et al., 2023)	Tier 3: Mechanically relevant clonal geometry; causal forces unmeasured

Note. Marker expression does not establish a uniform lineage identity across regions or models. Mechanical-response evidence is reported only when directly tested; otherwise the relevant gap is stated.

### 2.2 Basement membrane and interstitial ECM define the epithelial–stromal interface

#### 2.2.1 Layer-specific matrix organization constrains gland architecture

The gland–stroma interface is organized as connected but mechanically non-equivalent compartments. The apical surface faces the lumen, while apical and lateral junctions distribute tension across neighboring epithelial cells. Basally, laminin- and type IV collagen-rich basement membrane supports polarity and selective attachment through integrin-containing adhesions. Outside this boundary, the lamina propria contains fibrillar collagens, fibronectin, proteoglycans, and other matricellular proteins together with fibroblasts, immune cells, capillaries, nerves, and interstitial fluid. Thus, force can be redistributed through at least three coupled routes—cell–cell junctions, epithelial–basement-membrane adhesions, and the interstitial stromal network—whose composition and scale differ.

A change in one compartment cannot be assumed to produce the same material change in another. Protease activity may interrupt a basement-membrane boundary without increasing bulk stiffness. Collagen accumulation may change fiber spacing, transport, ligand presentation, and resistance to deformation, whereas edema may change tissue volume without a parallel rise in solid-matrix modulus. Conversely, a laminin-rich attachment interface may persist even when gland shape and the surrounding interstitium are abnormal. Human intestinal metaplasia and carcinoma show a persistent laminin–α6β4-integrin interface, whereas *H. pylori*-associated gastritis alters MMP/ADAM remodeling networks (Sampieri, 2013; Tani et al., 1996). Figure 1B therefore maps the wall from lumen to epithelium, basement membrane, and lamina propria so that every illustrated interaction has an anatomically defined source and recipient.

Regional ECM specialization remains much less resolved than epithelial specialization. Available human studies establish selected adhesion and remodeling patterns, but they do not provide a complete corpus-versus-antrum matrisome or a gland-axis map of material properties. The revised anatomical framework therefore distinguishes what is known from what is being proposed: epithelial and stromal compartments are drawn explicitly, whereas any region-specific difference in modulus, viscoelasticity, residual stress, or dynamic strain is labeled as an unresolved measurement question. This boundary is important because an anatomically plausible force path is not equivalent to a measured force distribution.

#### 2.2.2 Adhesion complexes integrate ligand identity with force transmission

Integrins respond to ligand identity and density while also participating in force-dependent focal-adhesion maturation. ITGA2, which encodes the α2 integrin collagen receptor, is directly regulated by miR-30a in models of SPEM, intestinal-type early gastric carcinogenesis, and human precancerous progression (Min et al., 2020). This creates a plausible route by which a lineage-associated regulatory change could alter cellular responsiveness to collagen. However, the study did not independently manipulate collagen stiffness, adhesion force, or matrix mechanics; its mechanobiological interpretation remains a testable extension of the demonstrated miR-30a–ITGA2 relationship.

Increasing collagen-matrix density altered focal-adhesion signaling and disrupted the E-cadherin/beta-catenin complex in gastric cancer cells (Jang et al., 2018). Because density simultaneously changes ligand availability, pore structure, transport, and effective mechanics, this is not a pure stiffness experiment. Together with human spatial and genetic observations, the result supports altered structural information transfer while leaving force magnitude unresolved.

### 2.3 From gland geometry to baseline force transmission

Normal force transmission begins with geometry and attachment rather than with a single signaling molecule. Luminal pressure and epithelial secretion load the apical surface; deformation and cell crowding are redistributed through lateral junctions; basal integrins couple epithelial traction to the basement membrane; and the lamina propria distributes stress through fibrillar matrix, stromal cells, vessels, and interstitial fluid. Gland curvature, pit depth, neck diameter, and branching could therefore influence the localization of strain even when a bulk modulus is unchanged. These are mechanically plausible consequences of gland architecture, not direct measurements in normal human mucosa. Figure 1 separates the anatomical map from the proposed force paths to prevent that inference from being overstated.

Direct measurements that resolve this force path in normal human corpus and antral glands are not available. Atrophy, infection, and regeneration studies establish that epithelial responses depend on region, injury, and timing (Lim and Chung, 2023; Nagata et al., 2012; Oh et al., 2006), but they do not quantify epithelial tension or gland-level load. Section 4 therefore discusses integrin–FAK/Src, Hippo–YAP/TAZ, and Piezo1 only after the matrix and stage-specific remodeling have been defined.

### 2.4 Multiscale force balance across epithelial, matrix, and glandular compartments

Gastric maintenance spans molecular junctions and receptors, cellular tension and polarity, clonal gland competition and fission (McDonald et al., 2008), and tissue-level stromal, immune, neural, endocrine, and vascular signals (Li K. et al., 2024; Zhang T. and Tang, 2026; Zheng et al., 2026). Mechanical homeostasis is therefore not one optimal stiffness value but a range of structural and signaling states compatible with restoration of gland architecture.

Within that range, the same structural or signaling component can support restitution in one context and persistence of an abnormal state in another. Interpretation therefore requires the endpoint that matters biologically: restoration of the original gland architecture, stable lineage conversion, or expansion of an altered clone. Timing is one determinant, but it must be considered together with region, cell identity, inflammatory context, and reversibility. The pathway-specific evidence for this conditional behavior is evaluated in Sections 4 and 5 rather than repeated here.

Few studies measure local mechanics, ECM architecture, adhesion, lineage identity, and pathway activity in the same gastric region. Normal reference ranges for stiffness, viscoelasticity, residual stress, and force transmission therefore remain undefined, making coordinated measurement—not another single-marker comparison—the central experimental need.

### 2.5 Dynamic gastric loading and region-specific mechanical context

Gastric filling, wall motion, pressure variation, and fluid movement make cyclic strain and luminal shear plausible physiological inputs. However, the retained gastric-precancer literature did not quantify these exposures at the epithelial surface together with lineage or mechanotransduction outcomes. Dynamic loading is therefore presented here as a future experimental variable, not as a demonstrated driver of atrophy, metaplasia, or dysplasia. Its value is that loading rate, strain amplitude, stress relaxation, membrane tension, and calcium entry can be manipulated separately from static matrix composition.

Corpus and antral glands differ in architecture, epithelial composition, endocrine exposure, and lesion pattern. Whether they also differ reproducibly in local modulus, viscoelasticity, residual stress, or dynamic strain remains unknown; anatomical heterogeneity should not be mistaken for demonstrated mechanical heterogeneity. Porcine ultrasound elastography and biaxial testing nevertheless show pronounced layer-, region-, and direction-dependent behavior (Bauer et al., 2020; Dargar et al., 2019), explaining why a single bulk modulus is an inadequate physiological reference. A decisive human study would obtain matched antral and corpus samples from the same patient, register pathology and tissue layer, and measure static and time-dependent properties using the same protocol.

A future test should compare static and dynamically loaded patient-derived epithelial–stromal systems while independently controlling stiffness, viscoelasticity, ligand density, and degradability. Exposure ranges should first be derived from gastric measurements rather than chosen arbitrarily. Time-resolved lineage, calcium, YAP, and recovery readouts would then determine whether cyclic strain or shear changes response amplitude, direction, or reversibility. Until such studies are completed, dynamic loading should not be drawn as an established precancer pathway.

### 2.6 Mechanistic synthesis: from homeostasis to chronic injury

The healthy gastric mucosa is maintained by region-specific and injury-responsive stem/progenitor populations embedded within a layered adhesion and ECM system. Clonal gland organization operates at one scale, epithelial junctions and integrin attachments at another, and stromal, vascular, and luminal loading at the tissue scale. Current evidence establishes the biological relevance of these compartments but does not define a single mechanical set point for gastric homeostasis. This distinction provides the reference state for interpreting chronic injury without prematurely assigning a specific mechanotransduction pathway to every structural change.

This baseline prepares the central comparison developed in the next chapter. Chronic *H. pylori* infection, inflammation, atrophy, and metaplastic remodeling alter ECM turnover, collagen architecture, basement-membrane organization, epithelial adhesion, and stromal-cell behavior. The next section therefore examines how the normal dynamic range described here is progressively displaced during gastric precancerous progression—and which of those changes represent observed structure, inferred mechanics, or directly measured physical properties.

## 3 Stage-dependent remodeling of the gastric precancer mechanical niche

Chapter 2 defined gastric homeostasis as a dynamic balance among gland architecture, region-specific epithelial populations, adhesion systems, and context-dependent regenerative signals. Chronic injury displaces that balance. The resulting tissue is not characterized by a single matrix change; rather, synthesis, degradation, organization, cellular attachment, and stromal activity evolve together. This chapter separates those processes and distinguishes observed structural remodeling from inferred or directly measured mechanics.

### 3.1 Chronic injury coordinates epithelial, stromal, and matrix remodeling

Chronic *H. pylori*-associated injury provides the clearest upstream link to matrix remodeling. Infection alters epithelial and acid-secretory physiology and recruits inflammatory and stromal responses (Fu et al., 2026; Hao et al., 2026; Smolka and Backert, 2012). MMPs, their inhibitors, and related disintegrin-metalloproteases are altered during *H. pylori*-associated gastritis, providing direct evidence that basement-membrane and interstitial-ECM turnover can become persistently unbalanced (Sampieri, 2013). Infection is therefore treated as an upstream ecological perturbation that can initiate epithelial injury and inflammatory-stromal remodeling; it is not a mechanical input in itself.

The consequences of H. pylori-associated remodeling depend on injury duration, gastric region, host and bacterial characteristics, and the cells that dominate a lesion. Repeated epithelial loss increases demand on fibroblasts, mesenchymal stromal cells, immune cells, and regenerating epithelium. Repair may restore gland architecture, whereas persistent injury can maintain an imbalance between matrix production and proteolysis. Experimental interventions that improve atrophic-gastritis phenotypes or progenitor function support partial biological reversibility (Chen et al., 2024; Li K. et al., 2024; Xu et al., 2023), but they do not show that established metaplasia can be mechanically normalized. Conversely, the proposition that an altered mechanical niche sustains *H. pylori* colonization has not been directly tested and should not be used to close a bidirectional causal loop.

### 3.2 Matrix turnover and fiber organization evolve across pathological stages

Directly relevant structural observations remain limited and context dependent. Case-based observations and clinicopathological studies of collagenous gastritis document conspicuous subepithelial collagen deposition (Li et al., 2025; Ma et al., 2015; Sun et al., 2026; Winslow et al., 2001), but this uncommon condition cannot represent the usual atrophy–metaplasia sequence. Its relevance is illustrative: collagen accumulation can coexist with epithelial and inflammatory abnormalities without establishing a general mechanical trajectory for gastric precancer.

A layer-by-layer account is more informative than a list of ECM markers. In normal mucosa, a continuous epithelial basement membrane and balanced interstitial turnover preserve gland polarity and stromal spacing. During chronic gastritis and atrophy, gland loss, inflammatory infiltration, edema, fibroblast activation, and altered MMP/ADAM activity can change both the amount and organization of matrix around surviving glands (Sampieri, 2013). These changes could generate opposing local effects—for example, reduced attachment at a disrupted basement membrane beside increased fiber density or contractile stress in the lamina propria—but the cited histological studies did not measure those effects. They are therefore competing mechanical interpretations, not reported observations.

Metaplastic glands can establish an alternative epithelial–matrix interface. Human tissue shows laminin and α6β4-integrin patterns in intestinal metaplasia that resemble the attachment program later retained by intestinal-type carcinoma, indicating reorganization of ligand–receptor topology before invasion (Tani et al., 1996). Dysplasia adds epithelial crowding, loss of regular polarity, irregular gland curvature, and spatially heterogeneous stromal change. Optical studies detect stage-associated differences in gland morphology and collagen-related signals (Li et al., 2018; Li et al., 2019), whereas tenascin expression varies across inflammatory, dysplastic, and neoplastic gastric lesions (Tiitta et al., 1994). These observations support stage- and compartment-dependent remodeling; none establishes that every layer becomes progressively stiffer.

The same structural feature can have different mechanical implications depending on its location. Basement-membrane discontinuity may reduce local attachment, a nearby collagen-rich stromal focus may resist deformation, and epithelial crowding may generate cell-derived stress even if interstitial modulus is unchanged. Figure 2 places normal mucosa, atrophy, metaplasia, and dysplasia on the same anatomical coordinates—gland shape, basement-membrane continuity, interstitial fibers, turnover, and inflammatory/stromal context—so that differences can be compared without assigning an invented stiffness value.

**FIGURE 2:**
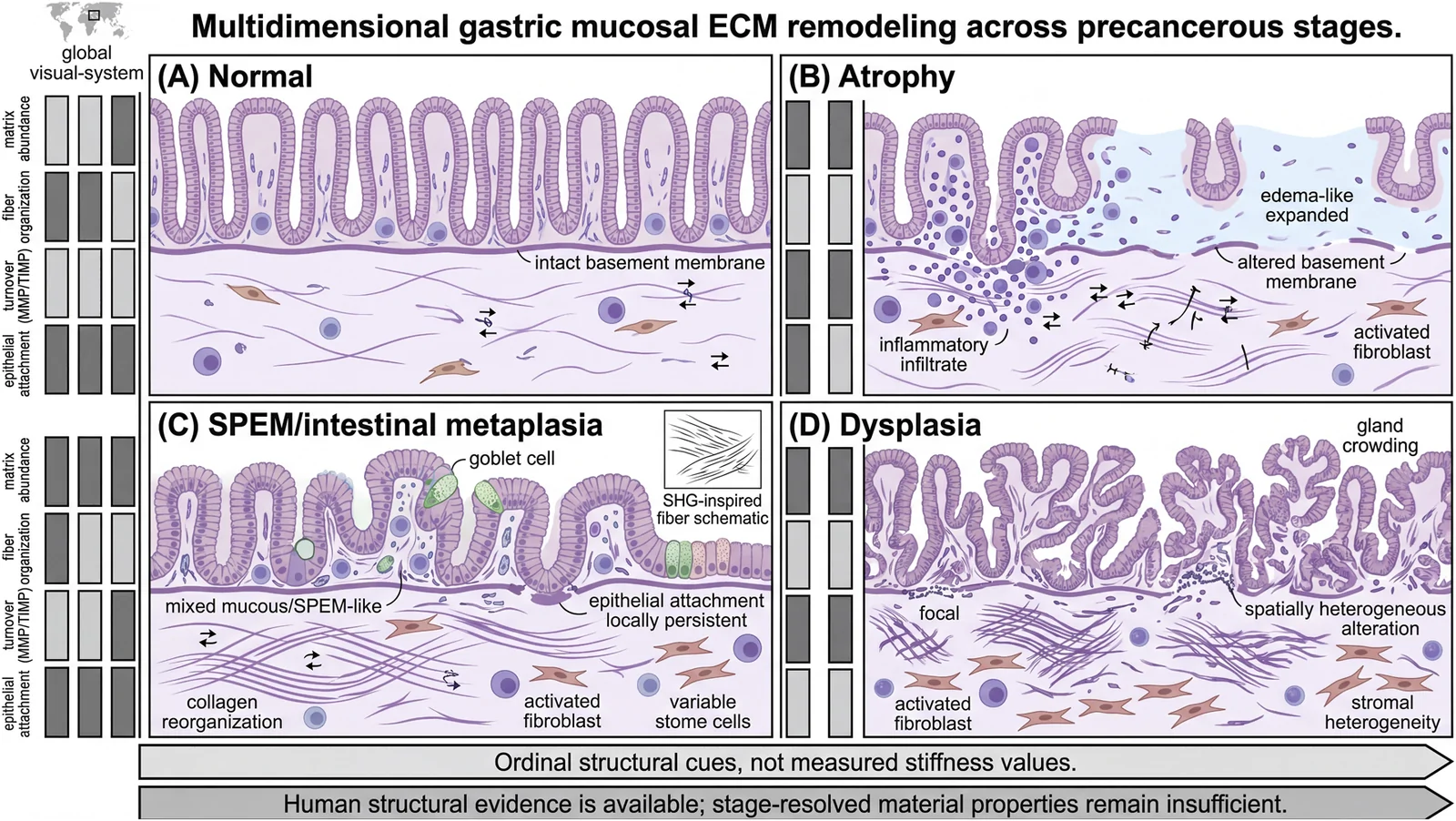
Stage-dependent remodeling of extracellular matrix and tissue architecture. **(A‐D)** compare normal mucosa, atrophy, SPEM/intestinal metaplasia, and dysplasia. Each panel separates gland organization, basement-membrane continuity, interstitial fiber architecture, matrix turnover, epithelial attachment, and inflammatory/stromal context. Side bars are ordinal visual summaries of reported structural evidence, not measured mechanical values. The figure does not assign quantitative stiffness, assume that every lesion follows the same sequence, or represent dynamic peristaltic loading. Abbreviations: ECM, extracellular matrix; IM, intestinal metaplasia; MMP, matrix metalloproteinase; SPEM, spasmolytic polypeptide-expressing metaplasia; TIMP, tissue inhibitor of metalloproteinases.

Multimodal nonlinear optical microscopy and second-harmonic generation visualize gland morphology and collagen architecture in premalignant gastric tissue (Li et al., 2018; Li et al., 2019). Under GPMES these are Tier 2 structural proxies, not measurements of elastic modulus. Their strongest role is spatial phenotyping and biopsy guidance paired with a force-sensitive method.

Figure 2 is therefore a stage-resolved structural map rather than a mechanical trajectory. The ordered panels aid comparison, but neither their arrangement nor the ordinal side bars imply obligatory linear progression or uniform stiffening.

### 3.3 Loss of barrier integrity and adhesion alters epithelial force transmission

ECM remodeling becomes biologically consequential when it changes how epithelial cells attach to one another and to their substrate. The E-cadherin/β-catenin complex distributes forces across epithelial sheets and helps maintain gland polarity. In a collagen-based material system, increased ECM density disrupted this complex and altered focal-adhesion signaling in gastric cancer cells (Jang et al., 2018). The result offers functional evidence that matrix context can destabilize epithelial organization. However, collagen concentration simultaneously changes ligand density, pore structure, diffusion, and material behavior; the experiment should not be described as a pure stiffness study.

Genetic and regulatory lesions converge on the same interface. CLDN18–ARHGAP26 fusion impaired barrier function and reduced cell–cell and cell–matrix adhesion through a mechanism involving RHOA regulation (Yao et al., 2015). During early intestinal-type carcinogenesis, loss of miR-30a released ITGA2, linking a metaplasia-associated regulatory change to a collagen-binding integrin (Min et al., 2020). Human spatial studies further suggest that metaplastic and malignant cells can establish laminin–α6β4-dependent attachment while losing other basement-membrane components (Tani et al., 1996). Together, these observations indicate that progression need not involve simple detachment. Cells may exchange a homeostatic adhesion system for an alternative one that preserves survival while permitting altered morphology, motility, or invasion.

### 3.4 Direct mechanical measurements reveal an incomplete and non-uniform trajectory

GPMES Tier 1 evidence is small but not absent. AFM indentation of four H. pylori-positive and four negative pediatric biopsies reported a lower mean Young’s modulus in positive tissue (0.78 ± 0.82 versus 3.27 ± 2.83 kPa) (Deptuła et al., 2021). This infection-status comparison is pediatric, *ex vivo*, and not histologically staged; it cannot define an atrophy-metaplasia-dysplasia trajectory or a population reference range.

Optical coherence elastography examined eight *ex vivo* samples from three patients across normal, metaplastic, dysplastic, and early signet-ring-cell-carcinoma regions (Gonzales et al., 2026). Relative stress contrast tended to increase in diseased tissue, but group differences were not significant and the readout is not interchangeable with AFM-derived Young’s modulus. Atomic-force spectroscopy separately reported lower adhesion and contact stiffness in malignant than normal epithelium (Dedkov and Dedkova, 2010). These very small, cross-sectional studies demonstrate feasibility, not generalizable stage-specific mechanics.

Two additional human studies extend the technical range but primarily concern established cancer. Laser resonance frequency analysis distinguished tumor from normal regions in resected specimens from two patients, with significant differences in the vibration-derived score (Furube et al., 2025). An endoscopic ultrasonic tactile sensor found early gastric cancers harder than normal mucosa or gastric ulcers in 23 cancer patients and 7 ulcer controls, although it could not distinguish mucosal from deeper invasion (Sakai et al., 2000). Together, these studies support the feasibility of gastric mechanical contrast while leaving stage-resolved precancer values unresolved.

Supplementary Table S1 consolidates the available direct or near-direct human gastric mechanical measurements and separates quantitative modulus, relative stress, vibration, tactile, adhesion, and contact-stiffness readouts. These non-uniform findings generate different predictions for the competing models in Figure 3: a mechanical-driver model requires physical change to precede lineage transition; an epithelial-first model predicts that mechanical contrast emerges after epithelial-state change; and an inflammation-mediated model predicts regionally heterogeneous, bidirectional changes that track inflammatory activity. Cross-sectional measurements alone cannot discriminate among these alternatives.

**FIGURE 3:**
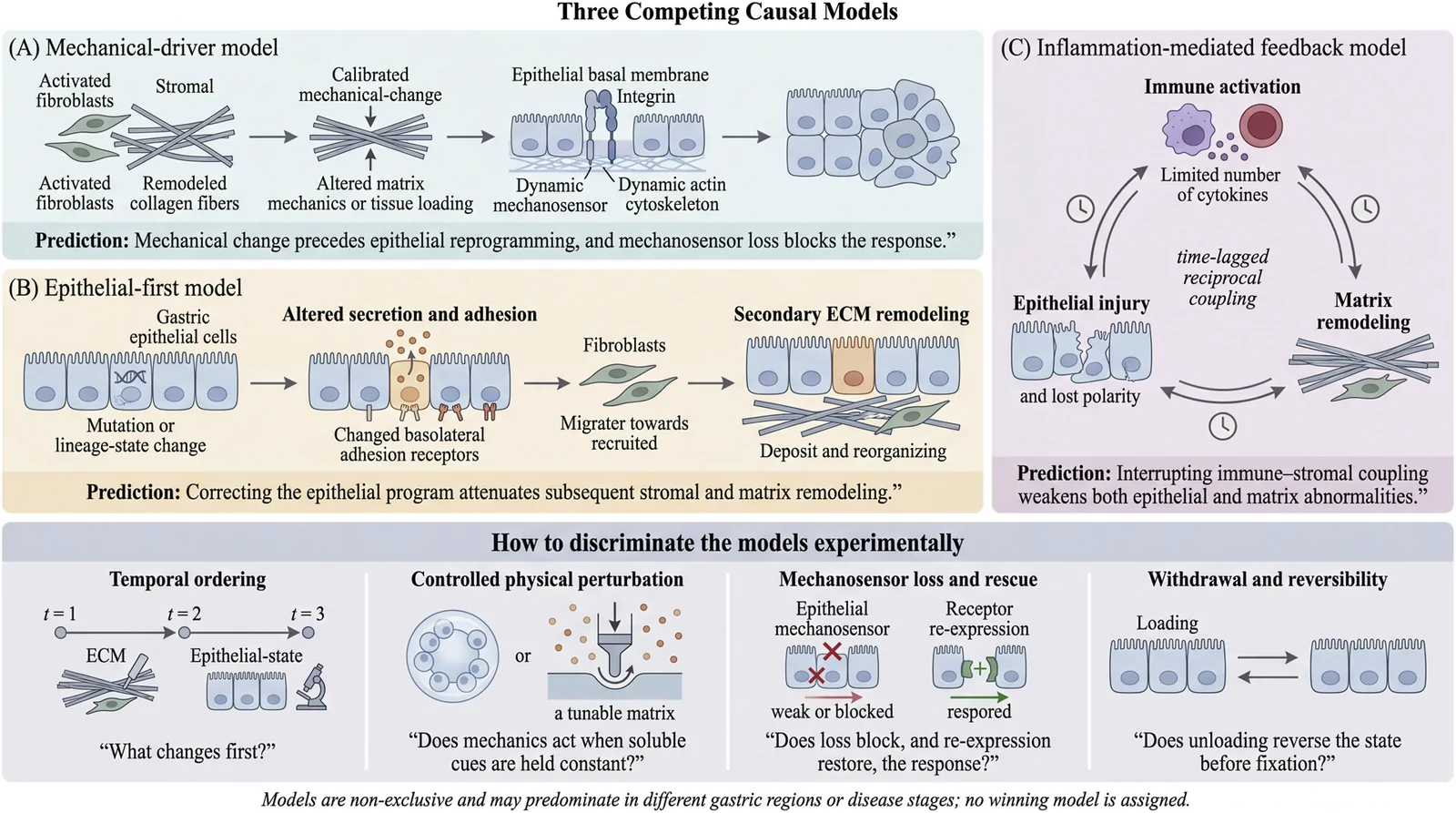
Competing causal models and discriminating experiments for the gastric precancer mechanical niche. **(A)** In the mechanical-driver model, a measured matrix or loading change precedes epithelial reprogramming, and the response should depend on the relevant mechanosensor and be reversible after mechanical rescue before fixation. **(B)** In the epithelial-first model, genetic or lineage change precedes fibroblast recruitment and secondary ECM remodeling; correction of the epithelial program should reduce later matrix change. **(C)** In the inflammation-mediated feedback model, immune activity, epithelial injury, and matrix remodeling show reciprocal time-lagged coupling, and interruption of inflammatory or stromal signaling should weaken both arms. The lower strip compares temporal ordering, independent physical perturbation, mechanosensor loss and rescue, and reversibility. Solid arrows indicate each model’s defining prediction; dashed arrows indicate unresolved feedback. The models are non-exclusive, and no model is presented as established causality.

Diagnostic and imaging studies detect molecular, glandular, or spectral abnormalities in high-grade intraepithelial neoplasia, gastritis, and dysplasia (El-Zimaity et al., 2018; Huang et al., 2023; Staibano et al., 2002; Wang et al., 2016). These approaches may improve lesion characterization, but none of the reported readouts should be interpreted as a direct measure of stiffness without calibration against an appropriate mechanical method.

The available literature supports substantial matrix and structural remodeling, but direct measurements of gastric precancer mechanics remain sparse. ECM abundance, fiber alignment, optical collagen signal, and mechanotransduction-marker expression are sometimes used as proxies, although none alone specifies stiffness, viscoelasticity, residual stress, interstitial pressure, or force transmission. Atomic-force microscopy or indentation can measure local resistance to deformation (Najera et al., 2023), rheological methods quantify bulk viscoelastic behavior (Forgacs et al., 1998), and Brillouin microscopy provides a non-contact optical mechanical contrast with its own interpretation requirements (Scarcelli and Yun, 2008). These methods interrogate different scales and material properties and are not interchangeable. Table 2 compares the measurement domains, outputs, spatial scales, and interpretation limits of the principal methods.

**TABLE 2:** Methods for structural and mechanical assessment of gastric tissue.

Method	Primary readout	Direct mechanical measurement?	Spatial scale	Principal value	Main limitation	References	Pathology applicability
Atomic-force microscopy/indentation	Local apparent modulus and force–displacement response	Yes	Cellular to tissue microscale	Spatially resolved mechanical mapping	Surface preparation, model dependence, *ex vivo* bias	(Najera et al., 2023)	Normal/infected biopsy and any stage accessible at the surface
Bulk or microscale rheology	Storage/loss moduli and viscoelastic response	Yes	Millimeter to bulk tissue	Quantitative time-dependent material behavior	Limited spatial and cellular resolution	(Forgacs et al., 1998)	Requires larger tissue; stage mixing is likely
SHG/multimodal nonlinear optical microscopy	Collagen-associated architecture and gland morphology	No	Micrometer-scale imaging	Label-free spatial phenotyping	Optical signal is not equivalent to stiffness	(Li et al., 2018; Li et al., 2019)	All stages for architecture; indirect mechanics
Brillouin microscopy	Optical proxy related to longitudinal mechanical response	Indirect	Micrometer-scale imaging	Non-contact three-dimensional mapping	Strong influence of hydration and interpretation model	(Scarcelli and Yun, 2008)	Emerging; direct gastric precancer validation limited
Tunable matrix/organoid platforms	Cellular response to controlled material context	Mechanical manipulation, depending on design	Cell to organoid	Tests causality under controlled conditions	Material variables may covary; simplified ecology	(Jang et al., 2018)	Organoid/model stages; not equivalent to human tissue

Note. AFM, rheology, optical elastography, Brillouin imaging, and structural microscopy interrogate different material properties and spatial scales; their outputs should not be converted or compared without calibration.

The next-generation of gastric studies should register structural imaging, force measurements, pathology, and cell-state markers within the same tissue coordinates. Until such data exist, “matrix remodeling” is the evidence-supported term, whereas “stiffening” should be reserved for studies that directly assess a relevant mechanical property.

### 3.5 Stage-dependent remodeling cannot be reduced to uniform tissue stiffening

Chronic injury remodels the gastric niche through coordinated changes in matrix turnover, fiber architecture, basement membrane, and epithelial adhesion. Structural and molecular remodeling is well documented, whereas the available direct measurements are too sparse and heterogeneous to define how material properties change across pathological stages. This distinction leads to the next question: how do gastric cells sense an altered physical context, and which signaling routes have sufficient causal support to connect remodeling with disease behavior?

## 4 Mechanosensing pathways linking matrix state to epithelial behavior

Mechanotransduction should be read as a causal sequence, not as a list of pathways. A defensible chain begins with a specified physical exposure or material context, identifies the molecular sensor or adhesion complex, demonstrates force transmission through the cytoskeleton or membrane, resolves the nuclear or ionic response, and links that response to a stage-relevant epithelial phenotype. Evidence is strongest when the physical variable is measured or manipulated independently of ligand concentration and soluble signaling, when loss of the proposed sensor blocks the response, and when rescue restores it. Integrin-FAK/Src-Rho, Hippo-YAP/TAZ, and Piezo1 satisfy different portions of this chain. Table 3 states explicitly whether a defined mechanical stimulus was applied in each gastric study, so pathway importance is not mistaken for proof of mechanical causality.

**TABLE 3:** Mechanotransduction pathways, defined mechanical stimulation, pathology context, evidence tier, and causal strength.

Evidence category	Molecule or axis	Defined mechanical stimulus applied?	Principal output	Disease stage/model	GPMES tier/causal strength	References
Pathway or sensor perturbation	miR-30a-ITGA2/focal adhesion	No - gene/target regulation; no calibrated force or stiffness	Adhesion, proliferation, migration/invasion	SPEM, human precancer and early gastric cancer	Tier 3: functional target validation; no direct mechanical manipulation	(Min et al., 2020)
Pathway or sensor perturbation	THBS4-integrin alpha2-AKT	No - stromal ligand/pathway perturbation	Angiogenesis	Gastric cancer models	Tier 4: genetic/pharmacological pathway support	(He et al., 2021)
Pathway or sensor perturbation	LATS2-YAP	No - infection and pathway loss-of-function; no defined load	Time-dependent EMT and intestinal metaplasia control	Cells, mouse and human mucosa	Tier 3: functional loss-of-function and temporal evidence	(Molina-Castro et al., 2020)
Pathway or sensor perturbation	YAP targetome	No - genetic injury model; no controlled mechanical input	SPEM and proliferative reprogramming	Genetic mouse, single-cell and human tissue	Tier 3: strong multilevel mechanistic evidence	(Loe et al., 2023)
Pathway or sensor perturbation	R-spondin-YAP	No - injury/infection and pathway intervention	Regeneration and metaplasia	Mouse models	Tier 3: functional pathway intervention	(Fischer et al., 2022)
Pathway or sensor perturbation	Piezo1-Ca2+-HIF-1alpha	No - channel gain/loss-of-function without calibrated external force	EMT, angiogenesis and omental metastasis	Advanced gastric cancer	Tier 4: gain/loss evidence; no defined external force	(Wang et al., 2021)
Material-context perturbation	Collagen-matrix concentration varied	Partial - material context altered, but stiffness, ligand density, porosity and transport covaried	Focal-adhesion and junctional remodeling	Gastric cancer cells	Tier 4: functional material-context evidence; not a pure stiffness experiment	(Jang et al., 2018)

### 4.1 Integrin–FAK/src–rho signaling converts adhesion into contractile responses

Before an adhesion pathway is interpreted as mechanotransduction, three possibilities must be separated: altered receptor or ligand abundance, biochemical activation of the pathway, and force-dependent maturation of an adhesion complex. Gastric organoid and fibrosis-associated studies show reduced dependence on cell–cell or cell–matrix adhesion and TGF-β1-associated adhesive remodeling during disease progression (Lv et al., 2010; Tong et al., 2023). These findings establish functional adhesion changes, but the initiating force and its magnitude were not defined.

Integrins couple extracellular ligands to focal adhesions, where FAK/Src-family signaling and Rho GTPases regulate actin organization, contractility, survival, and migration. Gastric studies identify several disease-relevant entry points. miR-30a regulation of ITGA2 links early intestinal-type carcinogenesis to a collagen receptor (Min et al., 2020). THBS4 produced in an H. pylori-associated mesenchymal-stromal context acts through integrin α2 and AKT to support angiogenic behavior (He et al., 2021). α6β4 integrin and laminin redistribution mark a reorganized epithelial–matrix interface (Tani et al., 1996), while CLDN18–ARHGAP26 fusion perturbs RHOA-associated adhesion and epithelial integrity (Yao et al., 2015).

These findings support an integrin–cytoskeletal axis but leave the initiating physical variable unresolved. Receptor abundance can change without a change in force, increased ligand availability can activate integrin signaling biochemically, and oncogenic signaling can raise contractility downstream of adhesion. Material-context experiments provide stronger support because changing collagen density alters focal-adhesion and junctional behavior (Jang et al., 2018), but density simultaneously changes stiffness, porosity, transport, and ligand presentation. A definitive gastric-precancer experiment would therefore vary stiffness independently of ligand density and degradability, quantify traction or junctional tension, demonstrate integrin/FAK dependence, and then test whether the epithelial lineage outcome is rescued when the mechanical exposure is withdrawn.

### 4.2 Hippo–YAP/TAZ–TEAD integrates signal duration, lineage, and tissue context

YAP and TAZ integrate cytoskeletal organization, cell density, shape, adhesion, and growth-factor signals. In the canonical Hippo cascade, MST1/2 and LATS1/2 restrain YAP/TAZ by phosphorylation; changes in Rho–F-actin tension can also influence nuclear localization and TEAD-dependent transcription (Zhang et al., 2019). Gastric evidence shows why this pathway cannot be reduced to “YAP on equals cancer.”

H. pylori-associated experiments define a regulated LATS2–YAP sequence: early nuclear YAP is followed by LATS2 induction, YAP phosphorylation, and restraint of epithelial–mesenchymal transition and intestinal-metaplasia-associated responses (Molina-Castro et al., 2020). In oxyntic injury, R-spondin-dependent YAP activity supports gland regeneration (Fischer et al., 2022), whereas genetic and multi-omic work links sustained epithelial YAP activity to atrophy, proliferation, and SPEM-associated transcription (Loe et al., 2023). These studies establish pathway dynamics and feedback, but not a force threshold. Under GPMES this is Tier 3 functional evidence. The competing models in Figure 3 make different temporal predictions: YAP should follow a measured physical perturbation in a mechanical-driver model, follow epithelial reprogramming in an epithelial-first model, and covary with inflammatory–matrix cycles in a feedback model.

By contrast, Tier 4 evidence extends Hippo-pathway output into vascular and invasive behavior. TAZ–TEAD4 promotes vasculogenic mimicry in gastric-cancer models (Zhang et al., 2022), and YAP supports the tumor-promoting function of gastric cancer-derived mesenchymal stem cells (Pan et al., 2017). Regulatory studies involving miR-365, curcumin, and curzerene associate suppression of YAP/TEAD-related programs with improvement of experimental precancer phenotypes (Zeng et al., 2026; Zhang T. et al., 2020; Zhou et al., 2025). Compound pleiotropy and the late disease stage of the TAZ and stromal studies limit pathway-specific and stage-specific inference.

### 4.3 Piezo1–Ca^2+^ signaling: strong tumor evidence, unresolved precancer function

Piezo1 is especially important because it offers a sensor-level test of mechanical causality rather than a downstream expression correlation. The current gastric evidence derives chiefly from advanced cancer, where gain- and loss-of-function affected Ca^2+^ influx, HIF-1α-associated signaling, epithelial–mesenchymal transition, angiogenesis, and omental metastasis (Wang et al., 2021). A stage-appropriate test should expose patient-derived atrophy, metaplasia, and dysplasia models to measured gastric loading waveforms while varying baseline matrix properties independently. Real-time calcium imaging should be paired with lineage-state, proliferation, and recovery endpoints; genetic Piezo1 loss, orthogonal channel inhibition, channel re-expression, and calcium-pathway rescue would establish sensor dependence. Temporal ordering would then distinguish whether Piezo1 activation precedes epithelial-state change, follows it, or oscillates with inflammatory feedback. Until such experiments are performed, Piezo1 remains a Tier 4 mechanistic candidate rather than an established precancer sensor.

### 4.4 Crosstalk couples mechanical inputs to inflammatory and growth-factor signaling

Biochemical convergence is a central interpretive problem because the same downstream node can be activated without a defined mechanical input. Infection-associated host responses and microbial variation shape early carcinogenesis (Kocak et al., 2020; Thorell et al., 2017), while transcriptomic, glycosylation, and histopathological studies identify stage-specific epithelial programs (Galamb et al., 2007; Gomes C. et al., 2013a; Solcia et al., 1996; Zhao et al., 2009). These data provide biological context for mechanosensitive pathways, but they do not identify stiffness, strain, shear, or pressure as the initiating exposure. Figure 4 consequently separates physical input, adhesion or sensor engagement, signal transmission, nuclear response, and epithelial outcome; a connection is called mechanically causal only when the physical variable and sensor dependence are tested.

**FIGURE 4:**
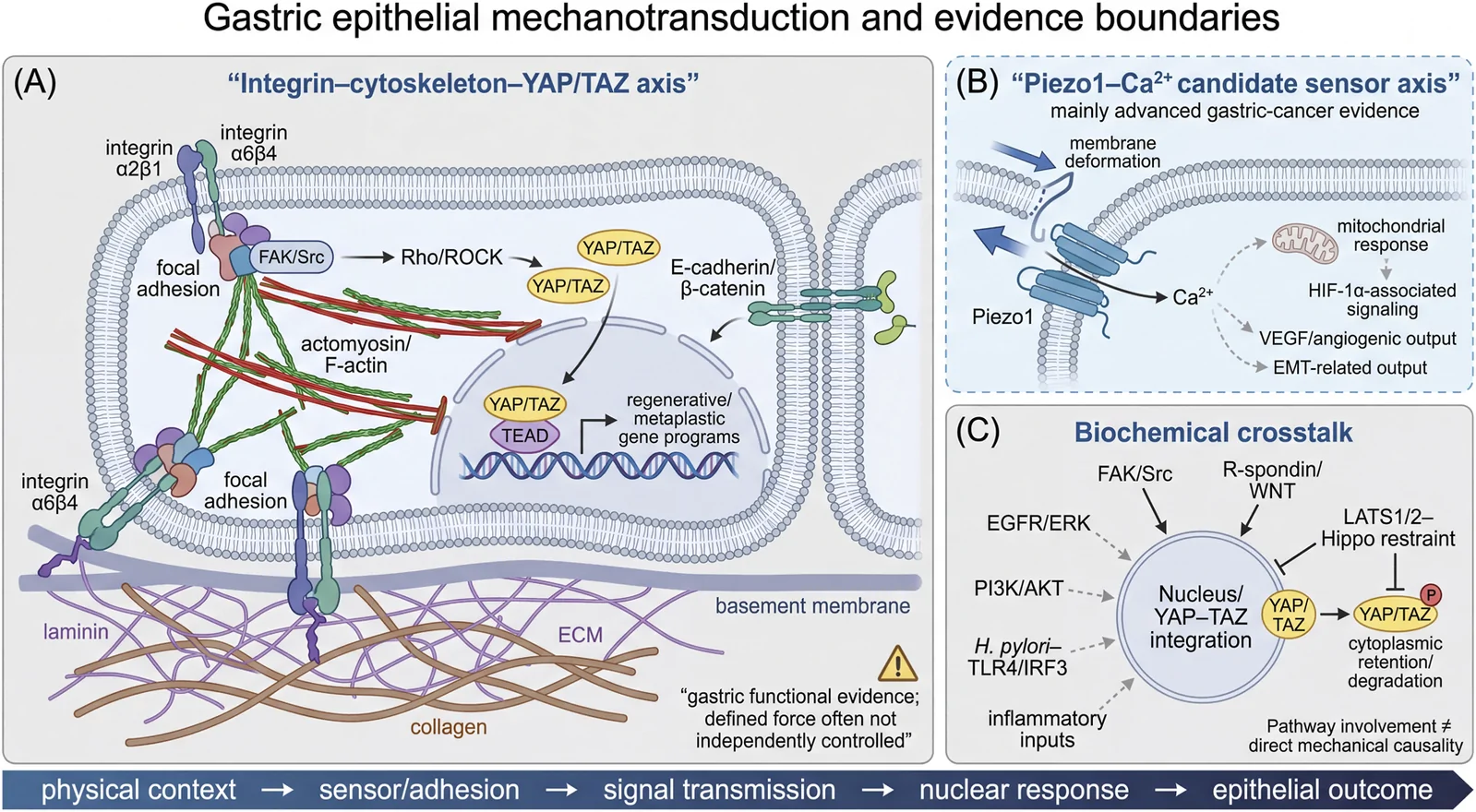
Mechanosensing, signal integration, and evidence boundaries. **(A)** The matrix–adhesion module distinguishes collagen–integrin α2β1 focal adhesions from laminin–integrin α6β4 hemidesmosome-like anchorage, then traces FAK/Src, Rho/ROCK, actomyosin tension, junctional coupling, and YAP/TAZ–TEAD output. **(B)** Piezo1-mediated Ca^2+^ entry and HIF-1α-associated metabolic or angiogenic outputs are isolated because the relevant gastric evidence derives mainly from advanced cancer. **(C)** Inflammatory and growth-factor pathways converge on YAP/TAZ without proving a common mechanical trigger. Solid arrows denote experimentally supported gastric functional links; dashed arrows denote advanced-cancer extrapolation or connections not demonstrated in human gastric precancer. Abbreviations: EMT, epithelial–mesenchymal transition; FAK, focal adhesion kinase; HIF-1α, hypoxia-inducible factor 1 alpha; TAZ, transcriptional coactivator with PDZ-binding motif; YAP, Yes-associated protein.

Mechanical signaling does not operate independently of biochemical inputs. EGFR–ERK/STAT signaling supports gastric progenitor renewal in atrophic-gastritis models (Li K. et al., 2024); PI3K/AKT is engaged downstream of integrin-related and matrix-associated pathways (He et al., 2021; Huang et al., 2026; Mao et al., 2026); TLR4/IRF3-associated regulation intersects with YAP in experimental precancer (Zhang T. et al., 2020); and HIF-1α links Piezo1-associated calcium responses to metabolic and angiogenic outputs (Wang et al., 2021). These intersections can create feedback: matrix remodeling changes adhesion and cytoskeletal state, transcriptional responses alter matrix production and proteolysis, and hypoxia or inflammation further reshapes both compartments.

Figure 4 separates this network into three modules that follow the same interpretive order. Panel A traces matrix ligand and integrin engagement through FAK/Src, Rho–actomyosin tension, and YAP/TAZ. Panel B isolates Piezo1–Ca^2+^–HIF-1α signaling and marks it as supported mainly by advanced gastric-cancer experiments. Panel C shows biochemical convergence without assigning a single upstream physical cause. Solid arrows are reserved for experimentally supported gastric links; dashed arrows indicate advanced-cancer extrapolation or a proposed connection that has not been demonstrated in human gastric precancer.

Note. In Table 3, ‘No’ indicates pathway, genetic, infection, or disease-context perturbation without a calibrated mechanical stimulus; ‘Partial’ indicates that a material context was altered while stiffness, ligand density, porosity, or transport also changed. Causal strength reflects the experimental design in the cited gastric study, not the general importance of the pathway.Advanced-cancer evidence is explicitly separated from precancer evidence.

### 4.5 Pathway hierarchy and evidence boundaries in gastric precancer

The most defensible mechanotransduction model is a hierarchy rather than a single pathway. Adhesion and focal-adhesion evidence is convergent but physically under-specified; YAP has strong functional evidence in gastric regeneration and metaplasia but context-dependent directionality; and Piezo1 provides a direct sensor candidate whose evidence is concentrated in advanced cancer. The next chapter asks how these signaling properties translate into epithelial fate decisions.

## 5 Mechanical regulation of epithelial plasticity, metaplasia, and clonal behavior

### 5.1 Mechanical context may shape stem-cell maintenance and clonal competition

The evidence for epithelial-state change answers three different questions. First, human molecular studies distinguish precancerous and early-cancer states, identify damage-response barriers in intestinal metaplasia, and localize intestinal stem-cell markers within metaplastic glands (Jang et al., 2015; Krishnan et al., 2020; Li F. et al., 2024; Zhang Y. et al., 2020). Second, perturbation studies show that CDX1 can impose an intestinal program through SALL4-and KLF5-associated reprogramming and that defined acute injuries can recruit chief-cell, isthmus, or competing epithelial routes depending on the model (Bockerstett et al., 2018; Fujii et al., 2012; Hata et al., 2020; Nam et al., 2010; Seishima et al., 2015). Third, eradication studies demonstrate reversibility of selected proliferative gland lesions but not universal reversal of established metaplasia (Nozaki et al., 2002). Clinical and conceptual syntheses likewise emphasize heterogeneity in cellular origin and premalignant significance (Busuttil and Boussioutas, 2009; Graham and Zou, 2018; Lai, 2019; Spechler et al., 2017). Barrett’s metaplasia provides a cross-organ example of hybrid epithelial states (Singh et al., 2021), not evidence for a gastric lineage relationship. Together, these findings support branching, model-dependent origins rather than a universal sequence.

Gastric precancer emerges in a tissue already organized into clonal gland units. Human mitochondrial lineage analysis shows that mutated stem-cell clones can undergo monoclonal conversion and spread through gland or crypt fission, including within intestinal metaplasia (McDonald et al., 2008). Fission therefore provides a tissue-level route by which a locally selected state can occupy a broader mucosal field. Its mechanical contribution remains unresolved: expansion requires changes in epithelial packing, gland curvature, basement-membrane continuity, and stromal accommodation, but the cited lineage studies did not measure force. Time-lapse organoid or *ex vivo* gland imaging combined with deformation or traction mapping could test whether mechanical constraints influence clone retention or merely accompany fission.

Experimental models further show that altered progenitor signaling changes disease trajectory. Targeted oncogenic K-ras activity in gastric progenitor cells produces chronic inflammation, microenvironmental change, and intraepithelial neoplasia (Okumura et al., 2010). Conversely, gastrin-dependent expansion of Cck2r-positive corpus progenitors promotes ulcer repair and restrains dysplasia in the reported model (Zheng et al., 2026). These studies demonstrate that progenitor expansion is not intrinsically oncogenic; its outcome depends on the initiating signal and niche context.

### 5.2 SPEM, intestinal metaplasia, and dysplasia represent branching lineage states

SPEM and intestinal metaplasia are branching injury-associated lineage states rather than interchangeable obligatory steps. Defined lineage experiments support chief-cell and isthmus-associated contributions under different injuries (Bockerstett et al., 2018; Nam et al., 2010), whereas human molecular and histopathological studies identify heterogeneous metaplastic programs and stage-specific epithelial states (Galamb et al., 2007; Li F. et al., 2024; Thorell et al., 2017). The evidence therefore supports more than one route into metaplasia and does not justify assigning all human lesions to a single cell of origin.

Mechanical influence should therefore be tested as a lineage-selection problem rather than assumed from co-occurrence. Under the mechanical-driver model, a controlled physical change should precede and alter the probability or persistence of a defined lineage transition. Under the epithelial-first model, matrix and force changes should appear only after reprogramming. Under the feedback model, epithelial state, inflammation, and matrix behavior should become mutually reinforcing and should respond incompletely to isolated perturbation. This framework prevents gland deformation, YAP activation, or ECM remodeling from being treated as proof of one universal SPEM-to-intestinal-metaplasia sequence.

YAP-centered studies clarify how signaling is translated into lineage outcomes. Experimental maintenance of epithelial YAP activity generated SPEM-associated chromatin and transcriptional programs in a genetic gastric model (Loe et al., 2023). LATS2 limits *H. pylori*-induced EMT and intestinal-metaplasia phenotypes (Molina-Castro et al., 2020), whereas R-spondin/YAP signaling contributes to both oxyntic regeneration and infection-associated metaplasia (Fischer et al., 2022). These findings support functional pathway involvement and suggest that lineage outcome depends on whether an injury-response program resolves, remains spatially restricted, or becomes stabilized across gland units. They do not define a mechanical force threshold or a single cellular origin for SPEM or intestinal metaplasia.

### 5.3 Signal duration and tissue context define the repair-to-neoplasia window

Longitudinal, experimental, clinical, and review evidence places epithelial plasticity within a longer process involving persistent risk after *H. pylori* eradication, metabolic or stemness programs, glandular progression, and niche selection (Cao et al., 2023; Feng et al., 2025; Liu et al., 2022; Myeong et al., 2025; Walker et al., 2019; Wang et al., 2009). These studies differ in design and do not support a single rate or route of progression; they instead identify factors that may influence whether an altered state persists or expands.

The apparent conflict between regeneration and progression is better framed as a tissue-state problem. Injury-responsive EGFR or Hippo signaling can replenish damaged epithelium (Fischer et al., 2022; Li K. et al., 2024; Loe et al., 2023), whereas continued stimulation in an inflamed and remodeled tissue may prevent complete redifferentiation. Once clonal alterations are established, the same adhesion and survival systems may favor expansion rather than restoration. Signal duration is therefore one variable among tissue stage, cell identity, and clonal context—not a stand-alone explanation.

Figure 5 summarizes this branching model and distinguishes reversible regenerative signaling from persistent plasticity without implying obligatory progression.

**FIGURE 5:**
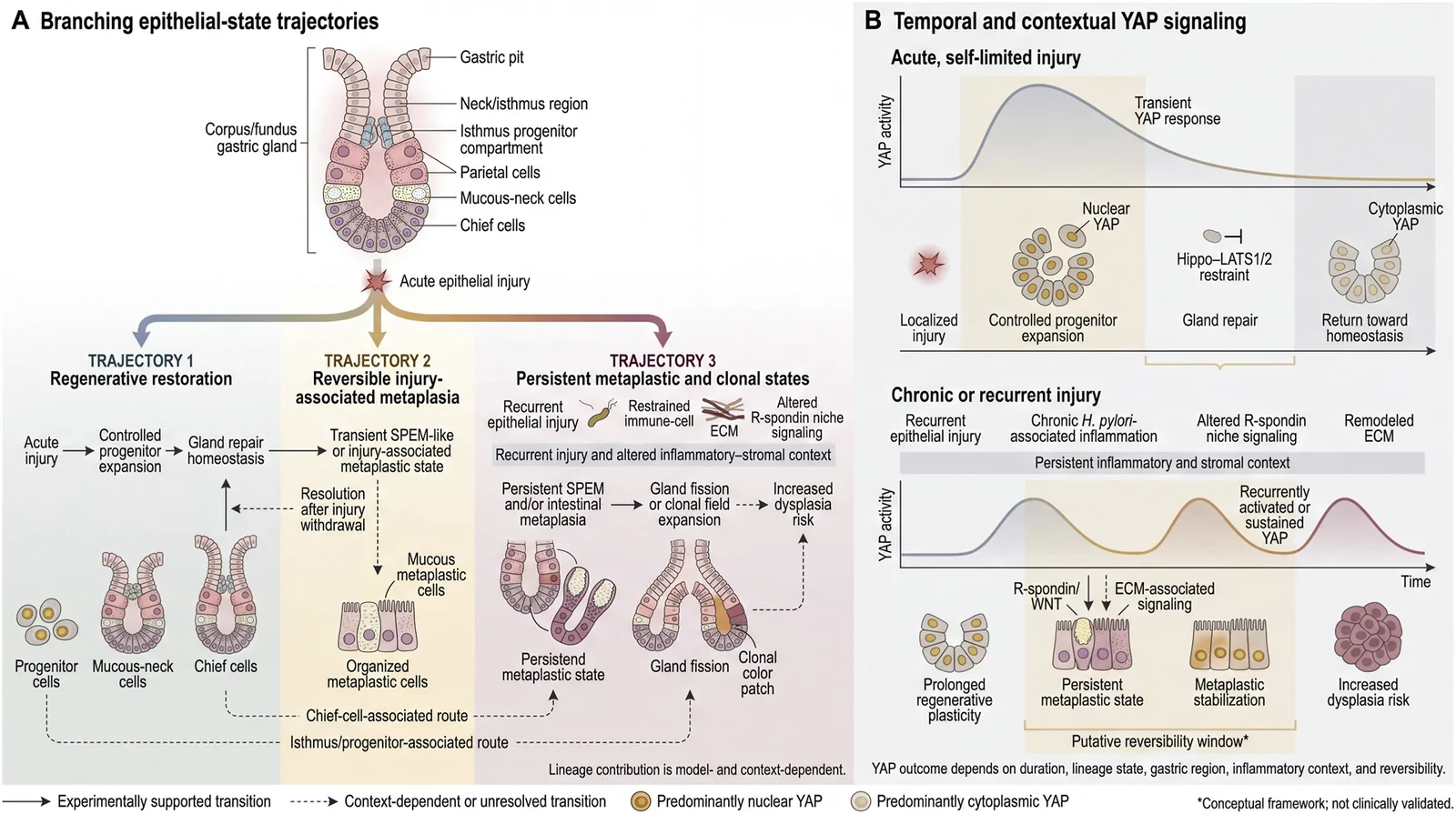
Epithelial-state transitions and context-dependent YAP signaling. **(A)** Homeostasis, injury-associated regeneration, SPEM, intestinal metaplasia, and dysplasia are displayed as branching states rather than an obligatory linear sequence. Arrows indicate experimentally supported lineage relationships only where established; unresolved transitions remain dashed. **(B)** Acute, self-limited injury is contrasted with chronic or recurrent injury. YAP localization and activity are interpreted together with lineage, spatial compartment, inflammatory state, and recovery after signal withdrawal. The panel therefore does not equate a brief YAP pulse with benefit or sustained YAP with harm in every context. Abbreviations: IM, intestinal metaplasia; SPEM, spasmolytic polypeptide-expressing metaplasia; YAP, Yes-associated protein.

### 5.4 Cell-fate consequences of persistent mechanical and inflammatory coupling

Cell fate is shaped by the interaction between lineage potential and tissue context. Regenerative programs become cancer-relevant when they fail to resolve, spread clonally, or operate in a matrix and inflammatory environment that rewards persistent plasticity. Because epithelial cells do not create that environment alone, the next chapter examines stromal, immune, metabolic, and vascular feedback.

## 6 Multicellular feedback reshapes the gastric precancer mechanical niche

### 6.1 Fibroblasts and mesenchymal stromal cells regulate matrix turnover and repair

Stromal effects depend on stromal state. In aging mice, human umbilical mesenchymal stromal cells improved atrophic-gastritis features through an Ndufs8-associated mitochondrial-autophagy mechanism, providing model-specific evidence for a reparative phenotype (Rui et al., 2024). Other experimental MSC interventions also improve selected histological or regenerative endpoints (Chen et al., 2024; Xu et al., 2023). These results cannot be generalized to endogenous stromal cells exposed to persistent inflammation or to tumor-conditioned MSCs, which may support different matrix and epithelial programs.

Chronic injury can redirect this compartment from repair toward persistent remodeling. Gastritis activates bone-marrow-derived mesenchymal stromal cells through TGF-β-associated signaling in mice, and CXCR4–SDF-1 contributes to acute gastric trafficking, whereas chronic recruitment becomes only partly CXCR4 dependent (Donnelly et al., 2014; Stoicov et al., 2013). *H. pylori* infection can also recruit bone-marrow-derived cells into preneoplastic lesions (Varon et al., 2012). In Figure 6, these signals originate in the lamina propria and reach basal or basolateral epithelial receptors across the gland–stroma interface; no stromal TGF-β route crosses the lumen. Advanced-cancer studies of MSC-derived THBS4 and YAP-associated programs are retained as Tier 4 evidence of possible stromal capability, not as proof of a precancer mechanism (He et al., 2021; Pan et al., 2017).

**FIGURE 6:**
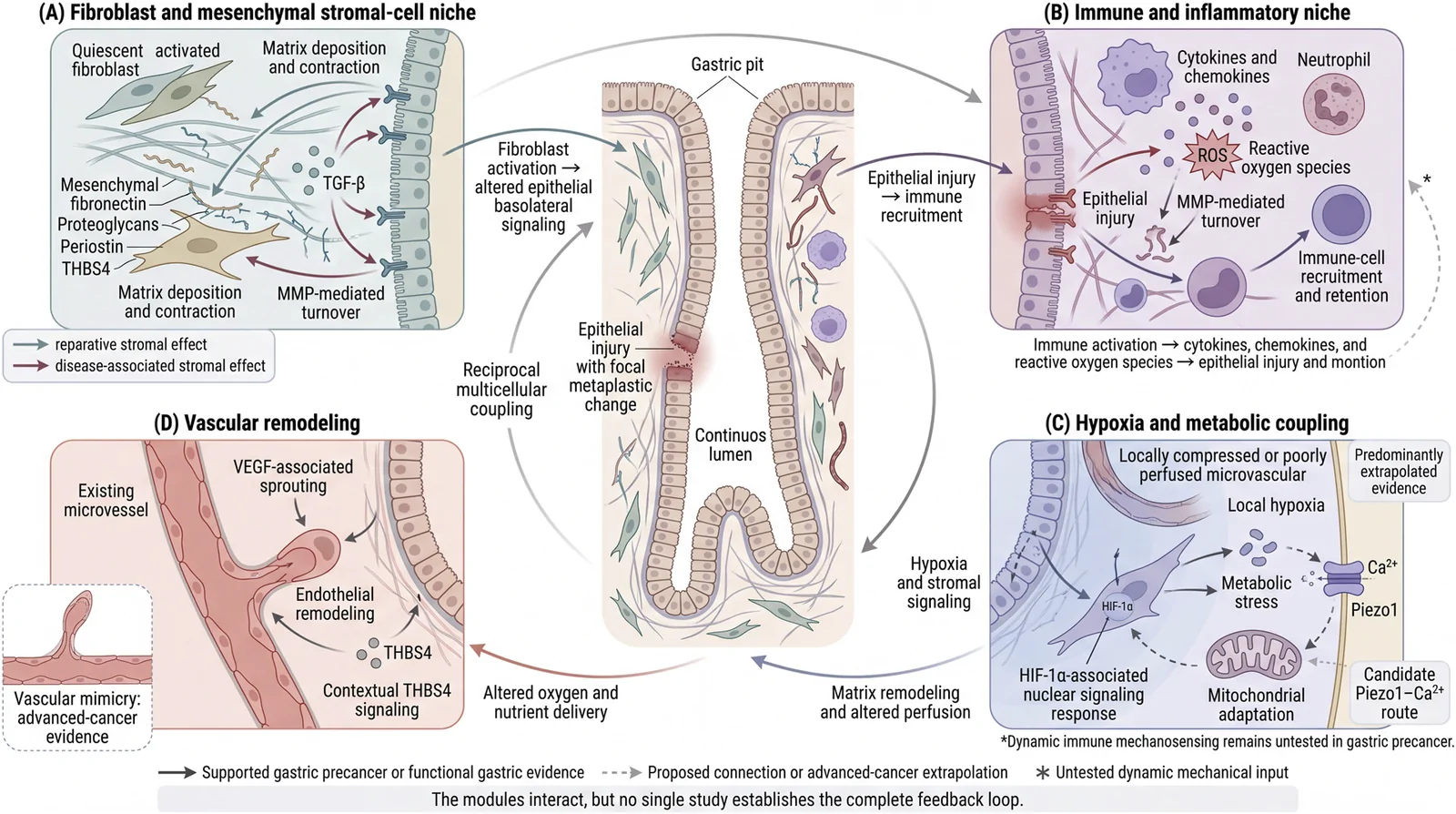
Multicellular feedback at the gastric gland–matrix interface. The central gland is oriented with the lumen and apical epithelial surface inside and the basement membrane and lamina propria outside. **(A)** Fibroblast and mesenchymal-stromal-cell states are contrasted across reparative and chronically conditioned contexts; matrix deposition, MMP-mediated turnover, and TGF-β signaling remain outside the gland. TGF-β reaches basal or basolateral epithelial receptors across the gland–stroma interface and is never shown entering from the lumen. **(B)** Immune cells and inflammatory mediators regulate epithelial injury and matrix turnover; direct dynamic immune mechanosensing in gastric precancer is labeled untested. **(C)** Hypoxic and metabolic responses are separated from the Piezo1–Ca^2+^ candidate route, whose precancer role remains unvalidated. **(D)** Vascular remodeling and vascular mimicry are marked as advanced-cancer or context-dependent evidence. Solid arrows denote gastric tissue or functional gastric evidence; dashed arrows denote proposed links or evidence extrapolated mainly from advanced gastric cancer. Abbreviations: ECM, extracellular matrix; GC, gastric cancer; MMP, matrix metalloproteinase; MSC, mesenchymal stromal cell; TGF-β, transforming growth factor beta; THBS4, thrombospondin 4.

### 6.2 Immune–matrix feedback couples chronic inflammation to tissue remodeling

Evidence for immune–matrix coupling is strongest for inflammatory remodeling and weaker for direct immune mechanosensing. In *H. pylori*-associated gastritis, altered MMP/ADAM networks connect inflammatory activity with matrix turnover (Sampieri, 2013), and eosinophil-rich or inflammatory-transformation studies show that lesions can contain distinct immune states (Fu et al., 2026; Li X. et al., 2026). By contrast, immune exclusion, stromal heterogeneity, and related multicellular patterns are documented mainly in established gastric cancer (Han et al., 2026; Khurram et al., 2025; Stancu et al., 2023); Tr1 responses likewise differ across infection and cancer contexts (Song et al., 2018). These observations justify testing reciprocal immune–matrix feedback, but none demonstrates that a defined force directly controls immune-cell behavior during human gastric precancer.

### 6.3 Metabolic stress, hypoxia, and vascular remodeling interact with matrix state

Matrix density, cell proliferation, inflammation, and perfusion jointly determine nutrient and oxygen availability. Hypoxia and metabolic reprogramming can in turn increase matrix production, angiogenic signaling, and cell plasticity. Piezo1-associated HIF-1α and VEGF responses provide a functional example in advanced gastric cancer (Wang et al., 2021). THBS4–integrin α2 signaling links stromal activity to angiogenesis (He et al., 2021), while MALAT1, angiogenesis-related lncRNAs, ADAMTS1, and TAZ–TEAD4 studies identify additional vascular or vasculogenic-mimicry programs (Chen et al., 2012; Han et al., 2021; Li et al., 2017; Zhang et al., 2022). Most of this evidence comes from established cancer and should be used to formulate, not confirm, a precancerous feedback model.

Figure 6 organizes the multicellular niche around an explicitly oriented gastric gland. The lumen and apical epithelial surface are inside the gland; the basement membrane and lamina propria are outside. Fibroblast/MSC, immune, vascular, and hypoxic-metabolic modules are arranged around that common coordinate so the source, recipient compartment, and direction of each signal can be read without crossing the lumen. The figure summarizes plausible feedback among compartments but does not imply that every arrow has been demonstrated in one model or at the same pathological stage.

### 6.4 Dynamic loading as a candidate regulator of immune-cell access

Matrix architecture, tissue deformation, and fluid movement could influence immune-cell access, residence, and activation, but direct gastric-precancer evidence is absent. A discriminating experiment would combine epithelial, stromal, and immune compartments under independently controlled matrix properties, cyclic strain, and luminal shear. Live tracking should separate recruitment from retention, while macrophage and lymphocyte activation states are measured beside epithelial injury and matrix turnover. Perturbing the physical variable without changing cytokine concentration—and then reversing the exposure—would test mechanical contribution; matched spatial human samples would determine whether the same cellular neighborhoods occur *in vivo*. Until these conditions are met, dynamic immune mechanosensing remains a hypothesis rather than an established feedback arm.

### 6.5 Multicellular feedback is a systems property rather than a linear pathway

The mechanical niche is therefore an emergent, spatially organized property rather than the output of one cell type or pathway. Epithelial injury changes stromal demand; stromal remodeling alters adhesion and transport; immune activity modifies both epithelial damage and proteolysis; and perfusion or metabolic stress can change the response of each compartment. Because compensation can occur across this network, a pathway inhibitor may suppress one readout while impairing repair or redirecting another compartment. Translational strategies must consequently measure tissue restoration, lesion control, and multicellular compensation together.

## 7 Translational implications of gastric precancer mechanobiology

### 7.1 Composite mechanical and molecular measurements for risk stratification

Published gastric biomarkers span tissue-associated proteins, serum proteins or glycoproteins, and multigene signatures (Eskandarion et al., 2024; Gomes C. et al., 2013b; Gong et al., 2024; Repetto et al., 2026; Wu et al., 2026). Their relevance here is as comparators, not as evidence of mechanics: none directly measures tissue material properties or establishes mechanically initiated signaling. A research-stage mechanical panel should therefore be evaluated for incremental value beyond histopathology, endoscopic findings, and established clinical factors, with atrophy, intestinal metaplasia, and dysplasia analyzed separately rather than pooled as one risk state.

The most plausible investigational biomarker is composite rather than singular. A structural or optical signature could be combined with epithelial-state markers and a restrained mechanotransduction panel. Atrophy studies should test reversibility by comparing matched biopsies before and after removal of the injurious stimulus and asking whether mechanical/structural measures recover with gland function. Metaplasia studies should quantify persistence, extent, and progression, whereas dysplasia studies should prioritize discrimination of immediate high-risk lesions. These designs can evaluate whether a mechanical feature adds prognostic information, but no present evidence supports stiffness, collagen organization, YAP localization, or Piezo1 as a threshold for surveillance or treatment.

### 7.2 Candidate intervention axes must preserve physiological repair

Potential intervention axes include matrix remodeling, integrin/FAK signaling, YAP/TEAD activity, Piezo1, and regenerative niche signals. Their safety cannot be inferred from disease stage alone. A preclinical stage-aware matrix is more defensible than a treatment algorithm: atrophy models should require restoration of gland function without persistent proliferation; metaplasia models should require resolution of abnormal lineage programs without impairing repair; and dysplasia models should require lesion control plus assessment of surrounding mucosa. Current evidence does not justify activating YAP with natural products in atrophy, prescribing integrin/FAK blockade in metaplasia, or combining Piezo1 and YAP inhibitors in dysplasia.

Intervention evidence is similarly heterogeneous and should be grouped by what it can establish. Paeonol, Weierning, calycosin, OLFM4-directed nanoparticles, and other multi-target or YAP-related regimens improve selected endpoints in distinct experimental settings (Han et al., 2023; Huang et al., 2026; Li D. et al., 2020; Li H. et al., 2020; Zeng et al., 2026; Zhang L. and Zou, 2026; Zhang T. et al., 2020; Zhou et al., 2025), while syndrome-associated transcriptomics characterizes molecular differences rather than testing a mechanical intervention (You et al., 2021). Pleiotropy, model dependence, and incomplete separation of repair from lesion suppression prevent attribution to a single mechanical pathway. MSC-based approaches require the additional distinction between administered reparative cells and chronically conditioned or tumor-derived stromal states (Chen et al., 2024; Donnelly et al., 2014; He et al., 2021; Pan et al., 2017; Stoicov et al., 2013; Varon et al., 2012; Xu et al., 2023). Table 4 therefore summarizes candidate axes and stage-specific validation requirements; it does not recommend clinical treatment.

**TABLE 4:** Candidate measurements and intervention axes: evidence stage, pathology context, and translational risk.

Candidate	Proposed use	GPMES tier/current evidence stage	Potential value	Principal risk or gap	References	Stage to test	Risk-stratification role
ECM architecture/NLOM signature	Imaging biomarker	Tier 2: Human *ex vivo* tissue	Spatial, label-free lesion characterization	Prospective and cross-center validation absent	(Li et al., 2018; Li et al., 2019; Tiitta et al., 1994)	Atrophy–dysplasia	Structural adjunct
Integrin/FAK-related profile	Biomarker or target	Tier 3–4: Precancer-to-cancer preclinical evidence	Links matrix recognition to cell behavior	May disrupt normal adhesion and repair	(He et al., 2021; Jang et al., 2018; Min et al., 2020)	Metaplasia–dysplasia	Candidate contextual marker
YAP/TAZ–TEAD	Stage-dependent biomarker or target	Tier 3–4: Strong preclinical gastric evidence	Integrates repair, metaplasia, and malignant behavior	Context-dependent; inhibition may impair regeneration	(Da, 2009; Fischer et al., 2022; Hu et al., 2014; Loe et al., 2023; Molina-Castro et al., 2020; Pan et al., 2017; Wang et al., 2025; Zeng et al., 2026; Zhang et al., 2019; Zhang et al., 2022; Zhang T. et al., 2020; Zhou et al., 2013; Zhou et al., 2025)	Metaplasia–dysplasia; stage specificity unresolved	Experimental pathway marker
Piezo1	Candidate sensor/target	Tier 4: Advanced gastric cancer	Mechanosensor-specific therapeutic route	Precancer function and mechanical activation unproven	(Wang et al., 2021)	Advanced-cancer evidence; precancer validation required	Not ready for risk use
Regenerative niche signals/MSC	Mucosal restoration	Tier 3: Experimental atrophy and gastritis	Potential reversal of regenerative failure	Dose/time-dependent proliferative and conditioning risks	(Chen et al., 2024; He et al., 2021; Li K. et al., 2024; Pan et al., 2017; Xu et al., 2023)	Atrophy–metaplasia	Repair-response marker
Composite mechano-molecular panel	Risk prediction	Not tiered: Conceptual	May capture structural and cellular heterogeneity	Requires prospective multicenter validation	(Jang et al., 2018; Li et al., 2018; Li et al., 2019; Loe et al., 2023; McDonald et al., 2008; Wang et al., 2021)	All stages as a composite research model	Conceptual integration only

Note. All entries are investigational. No candidate currently supports surveillance intervals or stage-specific treatment decisions without prospective validation.

### 7.3 Spatial mechanics and patient-derived models for translational validation

Patient-derived organoids provide a tractable platform for testing lineage and signaling responses, but common matrices have poorly controlled and batch-variable mechanical properties. Tunable synthetic or semi-synthetic hydrogels should independently control stiffness, viscoelasticity, ligand density, degradability, and fiber architecture. Co-culture with fibroblasts, immune cells, and endothelial cells can restore ecological complexity, while organ-on-chip systems can add flow, pressure, and repeated injury.

For human tissue, AFM or indentation should be spatially registered with SHG/NLOM, routine pathology, multiplex protein imaging, and spatial transcriptomic or proteomic measurements. Longitudinal sampling is essential because a single biopsy cannot distinguish transient repair from persistent pathological activation. Standardized reporting of tissue region, disease stage, preparation, hydration, loading rate, model fitting, and cellular composition will be necessary for cross-study comparison.

### 7.4 Translational readiness depends on stage-matched validation

Translational readiness requires agreement across three dimensions. The measurement must be analytically reproducible in a specified gastric region and tissue layer; the biological association must remain after adjustment for inflammation, gland composition, and disease stage; and prospective studies must show that the proposed marker or perturbation adds value beyond established histopathology and clinical risk factors. A candidate that meets only one dimension remains a research tool. Table 4 therefore presents pathology-specific validation requirements and risks rather than a treatment algorithm.

## 8 Discussion

### 8.1 An integrated model of stage- and context-dependent mechanobiology

The synthesis supports a bounded mechanical-niche framework. Chronic injury remodels gland architecture and matrix compartments; adhesion and cytoskeletal systems can translate parts of that context into epithelial-state signals; and epithelial, stromal, immune, metabolic, and vascular responses can feed back on the tissue environment. Figure 3 converts the remaining uncertainty into three non-exclusive causal models and four discriminating experimental tests. The framework is useful because it identifies where a physical measurement, a sensor-dependence test, or a temporal intervention could change inference. It does not assign one material property, one pathway, or one anatomical region as the universal controller of gastric precancer.

Two safeguards preserve that explanatory value. First, the Correa cascade is treated as a clinically useful progression framework that permits branching lineage and clonal trajectories rather than an obligatory cellular relay (McDonald et al., 2008; Zhang T. and Tang, 2026; Zhang T. et al., 2026). Second, ECM remodeling, pathway activation, and mechanical causality remain separate claims. GPMES operationalizes this distinction by ranking proximity to the gastric-precancer mechanics question—not general study quality—across direct human mechanics, human structural proxies, gastric functional perturbation, advanced-cancer capability, and cross-tissue analogy.

### 8.2 Competing causal models and evidence boundaries

As detailed in Section 3.4, the human measurement literature is too sparse and heterogeneous to infer a single stage-ordered material trajectory. Collagen architecture, AFM indentation, optical elastography, adhesion, and stress contrast are non-equivalent endpoints (Dedkov and Dedkova, 2010; Deptuła et al., 2021; Gonzales et al., 2026; Li et al., 2018; Li et al., 2019). The key datasets include only eight pediatric biopsies in one AFM comparison and eight *ex vivo* samples from three patients in the OCE series; neither supports population-level or stage-specific inference. Their disagreement instead motivates spatial co-registration of mechanics, pathology, matrix architecture, and cell composition within the same specimen.

YAP provides the clearest test of context dependence. Functional gastric studies place YAP in both regenerative and persistent metaplastic programs (Fischer et al., 2022; Loe et al., 2023; Molina-Castro et al., 2020), while pathology-based studies associate YAP expression with dysplasia, precancerous lesions, or carcinoma (Da, 2009; Hu et al., 2014; Zhou et al., 2013). The relevant prediction is not simply that short activation is beneficial and long activation harmful. After controlling for lineage, injury intensity, inflammatory signaling, and spatial extent, time-resolved YAP dynamics should predict whether a gland restores its original lineage, stabilizes metaplasia, or continues clonal expansion more accurately than a single endpoint measurement. Longitudinal reporters, timed perturbation, withdrawal, and rescue are required to test that prediction and to establish whether YAP is a mechanical mediator rather than a correlated injury response.

Figure 3 treats the mechanical-driver, epithelial-first, and inflammation-mediated feedback models as experimentally distinguishable rather than as alternative labels for the same sequence. A mechanical-driver model predicts that a measured matrix or loading change precedes epithelial reprogramming, that blocking the relevant mechanosensor prevents the lineage response despite persistence of the physical input, and that unloading or mechanical rescue can reverse the response before fixation. An epithelial-first model predicts that lineage or genetic change precedes stromal and ECM remodeling; correcting the epithelial program should then reduce secondary matrix change even without directly targeting the matrix.

The inflammation-mediated feedback model predicts reciprocal, time-lagged changes among immune activity, epithelial injury, and matrix remodeling. Interruption of inflammatory or stromal coupling should weaken both epithelial and mechanical abnormalities, whereas isolated mechanosensor inhibition may provide only partial rescue. These predictions require longitudinal measurement, independent physical perturbation, sensor loss-and-rescue, and withdrawal experiments. Cross-sectional co-localization cannot discriminate among the three models. The models may coexist across stages, and different gastric regions or spatially separate lesions within one patient may be dominated by different models at the same time.

*H. pylori* is best treated as an upstream perturbation rather than as a mechanical variable. It can enter the epithelial-first model through direct epithelial injury and the feedback model through coupled epithelial, immune, and stromal responses. A mechanically initiating route would require evidence that an infection-associated physical change occurs before epithelial reprogramming and remains necessary when soluble inflammatory signals are controlled; that evidence is not yet available.

### 8.3 Measurement and model constraints

Human tissue measurements are constrained by sampling and preparation. A gastric biopsy may contain normal glands, inflammation, atrophy, metaplasia, fibrosis, and edema, while orientation determines which tissue layer is loaded. Excision also releases residual stress and alters perfusion, hydration, and temperature. AFM results depend on surface preparation, indentation depth, probe geometry, and fitting model, whereas optical elastography measures a different mechanical contrast. These methods therefore require pathology registration and method-specific interpretation rather than direct numerical substitution.

Model systems answer complementary questions but differ in inferential reach. Cross-sectional human tissue defines spatial association; injury models and lineage tracing address temporal sequence; organoids and biomaterials permit controlled perturbation; and gastric-cancer models establish pathway capability in genetically selected cells. No single platform captures epithelium, stroma, immune cells, vasculature, innervation, and dynamic gastric loading. Convergent conclusions therefore require stage-matched findings across more than one model class.

### 8.4 Experimental priorities and criteria for translation

Translation should begin with measurement validity and stage-specific biological purpose rather than target nomination. Integrin–FAK, YAP/TAZ, Piezo1, and matrix-remodeling enzymes participate in physiological repair as well as disease-associated programs. A candidate intervention must therefore be tested at a defined lesion stage and exposure window, with simultaneous measurement of lesion control, restoration of normal gland function, multicellular compensation, and outcome after withdrawal. At present, these axes define experiments; they are not stage-specific therapeutic recommendations.

A clinically useful mechanical-niche biomarker requires three linked validations: analytical reproducibility across tissue handling and measurement platforms; biological co-registration with pathology, epithelial state, ECM composition, and multicellular context; and prospective evidence that a composite panel improves prediction beyond established histology and clinical risk factors. The same specimens should support these validation layers whenever possible.

A focused experimental sequence follows from these requirements. First, spatially co-register SHG or related matrix imaging with AFM, indentation, or calibrated optical elastography and pathology. Second, use patient-derived epithelial–stromal models to vary stiffness, viscoelasticity, ligand density, degradability, and physiologically justified dynamic loading independently. Third, apply time-resolved lineage tracing or clonal barcoding to determine whether mechanical perturbation changes state transition, clone persistence, gland fission, or resolution. Finally, integrate the same mechanical coordinates with spatial transcriptomic, proteomic, metabolic, and immune measurements. This workflow prioritizes causal discrimination over the accumulation of additional pathway correlations.

### 8.5 A mechanically extended correa cascade as a testable synthesis

A mechanically extended Correa cascade should be understood as three interacting axes rather than a new linear stage sequence. The inflammatory-injury axis alters epithelial turnover and stromal behavior; the physical-niche axis comprises matrix architecture, material behavior, adhesion, and dynamic loading; and the lineage–clonal axis determines whether glands recover, stabilize metaplasia, or expand dysplastic clones. The mechanical-driver, epithelial-first, and inflammation-mediated models differ in the temporal ordering among these axes. The synthesis is useful precisely because it yields competing predictions: physical change must lead lineage change in a driver model, lag behind it in an epithelial-first model, and show reciprocal time-dependent coupling in a feedback model.

## 9 Conclusion

Three principles emerge from this synthesis. First, GPMES makes inferential proximity explicit and prevents human mechanics, structural proxies, gastric perturbation models, advanced-cancer experiments, and cross-tissue analogy from being treated as equivalent evidence. Second, available human measurements are insufficient to define a stage-ordered material law; apparent behavior depends on pathological stage, gastric region, tissue layer, preparation, and measurement scale. Third, mechanotransduction outcome cannot be assigned from pathway expression alone: physical exposure, epithelial lineage, inflammatory context, signal dynamics, and reversibility must be resolved together.

The decisive next step is a linked measurement-and-perturbation program. Spatially registered human mechanics should define the exposure; dynamically loaded patient-derived models should test sensor dependence; and time-resolved lineage or clonal analysis with withdrawal and rescue should discriminate mechanical-driver, epithelial-first, and inflammation-mediated feedback models. This program converts the mechanical niche from a broad analogy into a falsifiable extension of the Correa cascade. Until those tests are completed, mechanical measurements and pathway markers should remain complementary research variables rather than stand-alone determinants of surveillance or treatment.
