# Supplementary material for: The mechanical niche in gastric precancer: extracellular matrix remodeling, mechanotransduction, and stem-cell fate
Source: Front Cell Dev Biol. 2026 Sep 28;14:1970363. doi: 10.3389/fcell.2026.1970363 (PMC13655520; doi:10.3389/fcell.2026.1970363)
Supplement: Supplementary file 1 [file Supplementaryfile1.docx]

# **Supplementary Table S1.** Direct and near-direct human gastric mechanical measurements identified in the evidence update. All included studies are classified as GPMES Tier 1 because they report direct or near-direct human mechanical contrasts; this designation does not remove method-specific limitations.

Note. Only human gastric measurements are listed. The table separates modulus, stress contrast, vibration, tactile hardness, adhesion, and contact stiffness because these endpoints are not interchangeable. Cancer studies establish feasibility but do not define the precancer trajectory.

| **Study** | **Samples; Disease comparison** | **Method/readout** | **Reported result** | **Key limitation** | **Reference** |
| --- | --- | --- | --- | --- | --- |
| Deptuła et al., 2021 | 4 H. pylori-negative and 4 positive pediatric biopsies; Infection status; not staged precancer | AFM indentation; Young’s modulus | 3.27 ± 2.83 vs 0.78 ± 0.82 kPa | Small, pediatric, ex vivo; histological stage not resolved | (Deptuła et al., 2021) |
| Gonzales et al., 2026 | 8 ex vivo samples from 3 patients; 17 OCE measurements; Normal, metaplasia, dysplasia, early cancer | OCT plus optical coherence elastography; stress contrast | Trend toward higher stress/relative stiffness in diseased tissue; groups not significantly different | Not a directly comparable Young’s modulus; ex vivo | (Gonzales et al., 2026) |
| Dedkov and Dedkova, 2010 | Excised normal and malignant human gastric epithelium; sample count incompletely reported; Normal versus malignant | Atomic-force spectroscopy; adhesion/contact stiffness | >10-fold decrease in adhesion and lower contact stiffness in malignant tissue | Cancer comparison; limited reporting; not precancer staged | (Dedkov and Dedkova, 2010) |
| Furube et al., 2025 | Resected gastric specimens from 2 patients; Normal versus gastric cancer regions | Laser resonance frequency analysis; vibration-derived score | Tumor and normal regions differed significantly in score (p = 0.00354) | Very small clinical series; indirect stiffness score; established cancer | (Furube et al., 2025) |
| Sakai et al., 2000 | 23 early gastric cancers and 7 active/healed gastric ulcers; Cancer versus normal mucosa or ulcer | Endoscopic ultrasonic tactile sensor; hardness index | Cancer was harder than normal mucosa or ulcer; invasion depth not discriminated | Older device; relative hardness; no precancer-stage comparison | (Sakai et al., 2000) |
